# Cyclin D2-knock-out mice with attenuated dentate gyrus neurogenesis have robust deficits in long-term memory formation

**DOI:** 10.1038/s41598-020-65090-1

**Published:** 2020-05-18

**Authors:** Stela P. Petkova, Michael Pride, Carolyn Klocke, Timothy A. Fenton, Jeannine White, Pamela J. Lein, Jacob Ellegood, Jason P. Lerch, Jill L. Silverman, Ben Waldau

**Affiliations:** 1 Department of Psychiatry and Behavioral Sciences, University of California Davis School of Medicine, Sacramento, CA 95817 US; 2 Department of Molecular Biosciences, UC Davis School of Veterinary Medicine, Davis, CA 95616 US; 3 Institute for Regenerative Cures, Sacramento, CA 95817 US; 4 MIND Institute, UC Davis, Sacramento, CA 95817 US; 5 Mouse Imaging Centre, Hospital for Sick Children, Toronto, Ontario M5T 3H7 Canada; 6 Wellcome Centre for Integrative Neuroimaging, FMRIB, Nuffield Department of Clinical Neuroscience,The University of Oxford, Oxford, OX3 9DU UK; 7 Department of Neurological Surgery, UC Davis Medical Center, Sacramento, CA 95817 US

**Keywords:** Neuroscience, Learning and memory, Hippocampus

## Abstract

Neurobehavioral studies have produced contradictory findings concerning the function of neurogenesis in the adult dentate gyrus. Previous studies have proved inconsistent across several behavioral endpoints thought to be dependent on dentate neurogenesis, including memory acquisition, short-term and long-term retention of memory, pattern separation, and reversal learning. We hypothesized that the main function of dentate neurogenesis is long-term memory formation because we assumed that a newly formed and integrated neuron would have a long-term impact on the local neural network. We used a cyclin D2-knock-out (cyclin D2^−/−^) mouse model of endogenously deficient dentate neurogenesis to test this hypothesis. We found that cyclin D2^−/−^ mice had robust and sustained loss of long-term memory in two separate behavioral tasks, Morris water maze (MWM) and touchscreen intermediate pattern separation. Moreover, after adjusting for differences in brain volumes determined by magnetic resonance (MR) imaging, reduced dentate neurogenesis moderately correlated with deficits in memory retention after 24 hours. Importantly, cyclin D2^−/−^ mice did not show deficits in learning acquisition in a touchscreen paradigm of intermediate pattern separation or MWM platform location, indicating intact short-term memory. Further evaluation of cyclin D2^−/−^ mice is necessary to confirm that deficits are specifically linked to dentate gyrus neurogenesis since cyclin D2^−/−^ mice also have a reduced size of the olfactory bulb, hippocampus, cerebellum and cortex besides reduced dentate gyrus neurogenesis.

## Introduction

The main function of neurogenesis in the adult dentate gyrus remains unknown. Currently, adult dentate neurogenesis has been implicated in pattern separation/completion^1–3^, deficits in spatial acquisition learning^4,5^, flexible learning/reversal learning^6,7^, fear learning^8^, novel object recognition^9^, and long-term memory formation^9,10^, while other authors have argued that it has no functional significance^11^.

Several critical reviews support pattern separation or higher resolution of memory as possible main functions of dentate neurogenesis^12–15^. Pattern separation describes a process in which similar memories are stored and retrieved as distinct memories. Experimental paradigms involving contextual fear discrimination learning of similar contexts^16,17^ and similar spatial locations^1^ have shown a role for hippocampal neurogenesis in pattern separation. However, experimental models require cautious interpretation because the perirhinal cortex and hippocampal CA3 regions, both of which form circuits with dentate neurons, have also been implicated in pattern separation^18,19^.

The data on long-term memory formation and dentate neurogenesis has been conflicting. Impaired performance in long-term memory tasks has been noted after irradiation or genetic ablation by several investigators^9,10,20^, but not by others^21,22^. In addition to impaired long-term memory retention in the Morris water maze, decreased neurogenesis has been linked to significant impairment in a hippocampus-dependent object recognition task after 3 hours, but not after 4 weeks^9^. Kitamura *et al*. demonstrated that attenuated neurogenesis promoted long-term potentiation (LTP) in rats^23^. On the other hand, Bruel-Jungerman *et al*. observed increased proliferation and survival of dentate progenitor cells with LTP^24^. Deng *et al*. found deficits in long-term memory retention 1 week after genetic ablation of dentate neurogenesis^25^.

We hypothesized that the main function of dentate neurogenesis is long-term memory formation based on the concept that a newly formed dentate neuron would be expected to have a lasting impact on the local network. We tested our hypothesis using a model of decreased dentate neurogenesis, the cyclin D2^−/−^ mouse. Compared to wild-type (WT) controls on the same genetic background, cyclin D2^−/−^ mice have nearly absent adult dentate neurogenesis^26^. While knockout animals have a similar body weight compared to WT, the cyclin D2^−/−^ phenotype also results in ovarian and testicular abnormalities^27^, altered humoral immunity^28^, and reduced size of the olfactory bulb, hippocampus, cerebellum and cortex^26^.

In the current study, we found no deficits in acquisition learning but loss of long-term memory retention in cyclin D2^−/−^ mice in two separate longitudinal behavioral tasks. We also saw a moderate correlation of the behavioral phenotype with reduced dentate neurogenesis adjusted for differences in brain volumes 24 hours after acquisition learning. Taken together, our data suggest that cyclin D2^−/−^ mice with attenuated dentate gyrus neurogenesis have robust deficits in long-term memory formation.

## Results

### Loss of cyclin D2 attenuates DG neurogenesis

**Figure 1:**
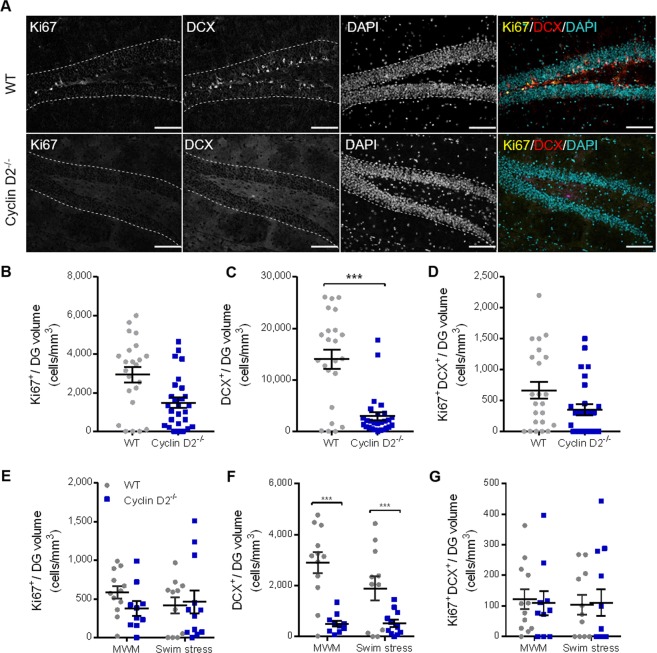
DG neurogenesis is attenuated in cyclin D2^−/−^ animals compared to cyclin D2^+/+^ (WT) controls regardless of behavioral experience. (**A**), Representative micrographs of WT and cyclin D2^−/−^ males in grayscale Ki67 (yellow), DCX (red), and DAPI (cyan) followed by a merge of all 3 channels at 20X magnification. Dashed lines indicate the outer boundary of the GCL; scale bars represent 200 μm. Total DG estimates of (**B**), Ki67^+^ cells, (**C**), DCX^+^ cells, and (**D**), Ki67^+^/DCX^+^ cells between WT (gray circles) and cyclin D2^−/−^ (blue squares) genotypes. (**E–G**), There was a significant main effect of genotype on total DG cell count estimates from panels **B–D** stratified across behavioral groups (MWM and forced swim stress). There was no statistically significant difference in neurogenesis between animals that underwent either MWM or swim stress, but a significant main effect of genotype remained. There was no statistically significant effect of sex, and the data are therefore combined by sex. Data represent mean ± SEM. Statistical outcome: *p ≤ 0.05, **p ≤ 0.01, ***p ≤ 0.0001 via one-way ANOVA (**B**-**D**, combined behavioral groups: N = 20–24/genotype) or multivariate ANOVA (**E**-**G**, stratified behavioral groups: N = 10–12/genotype/behavioral group).

We first verified the histological phenotype of cyclin D2^−/−^ mice (Fig. 1A). Global knockout of cyclin D2 reduced the number of Ki67^+^ cells in the DG, although this effect was not statistically significant (Fig. 1B). However, there was a significant decrease in DCX^+^ cells by 85.91% (95% CI: 80.58% − 91.24%) in cyclin D2^−/−^ animals compared to WT (ME of genotype, F_(3,40)_ = 11.70, p < 0.0001; t_40_ = 5.46, p < 0.0001; Fig. 1C). There was no difference between WT and cyclin D2^−/−^ genotypes in the number of Ki67^+^DCX^+^ double-positive cells in the DG (Fig. 1D). In comparing behavioral groups, there were no significant differences between MWM-trained animals and force swim stressed animals, although differences between genotypes remained statistically significant in DG DCX^+^ cells in both behavioral groups (MWM: F_(1,21)_ = 23.11, p < 0.0001; swim stress: F_(1,21)_ = 7.31, p = 0.014; Fig. 1E-G). Thus, MWM training and probing did not lead to an increase in neurogenesis compared to swim stress.

### MR imaging volumetric analysis

**Figure 2:**
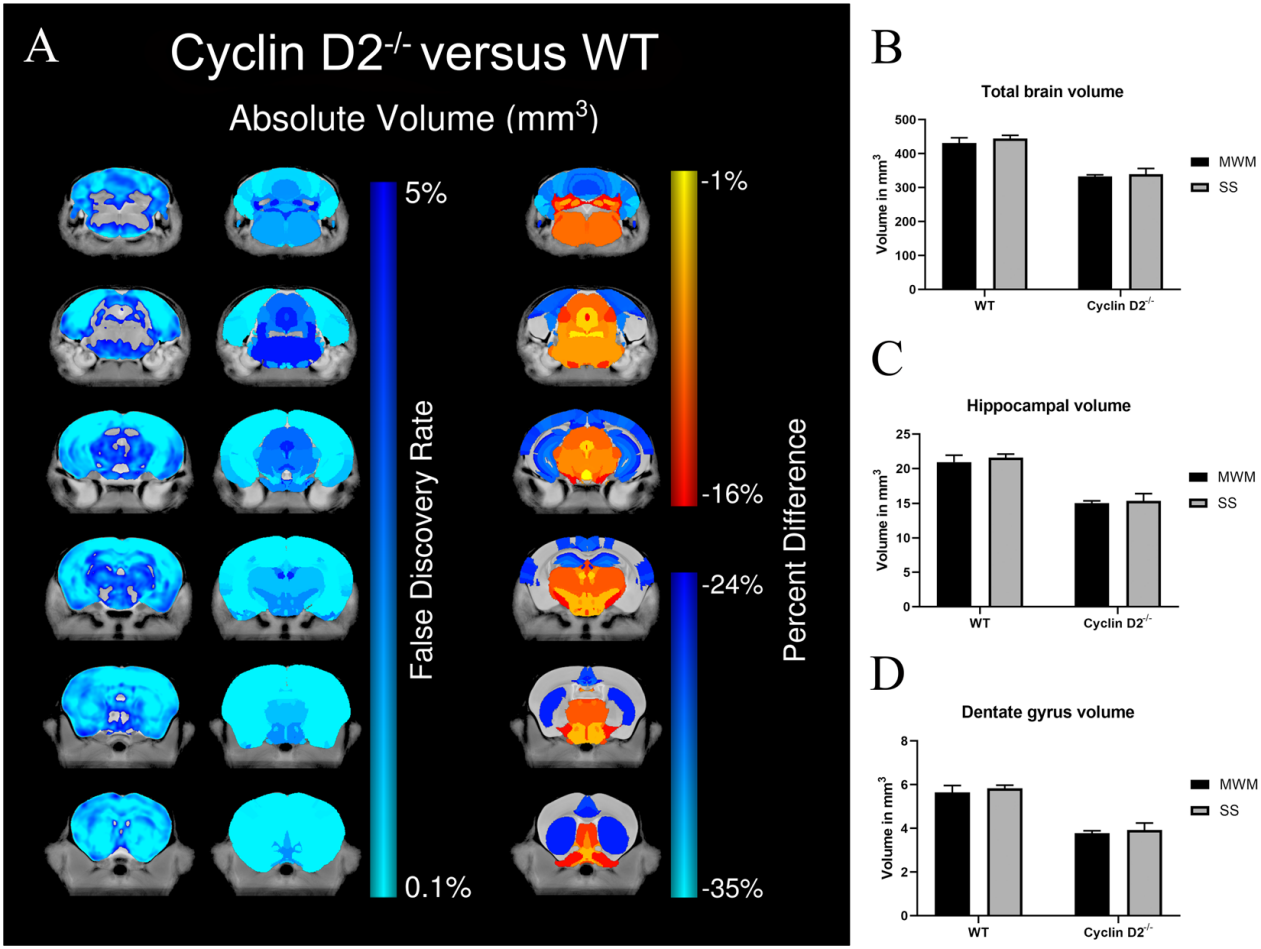
Cyclin D2^−/−^ mice have smaller brain volumes than WT, and volumes do not change when trained in Morris water maze. (**A**), Coronal slices highlighting both voxelwise (left) and regional percent (right) differences found in the cyclin D2^−/−^ mice when compared with their wild-type littermates. The cyclin D2^−/−^ mice were significantly smaller throughout the brain compared with WT mice (left); however, there was a stronger effect seen in both the cortex and the hippocampus (right), with a greater than 24% decrease in size for both structures. (**B–D**), Bar graphs highlighting the significant differences in absolute volume (mm^3^) found for total brain, hippocampal and DG volume. No significant differences were found in brain volumes between mice which have finished Morris water maze testing (MWM) versus swim stress (SS). Error bars represent 95% confidence intervals.

Since cyclin D2^−/−^ animals have smaller brains, which could impact behavioral results, we calculated anatomical differences between cyclin D2^−/−^ animals and WT animals with MRI (Fig. 2A). Cyclin D2^−/−^ animals had significantly smaller brain volumes than WT animals (333.31 mm^3^ versus 431.04 mm^3^; p < 0.0001, Fig. 2B). Their hippocampal formation (15.06 mm^3^ versus 20.94 mm^3^; p < 0.0001, Fig. 2C) and dentate gyri (3.78 mm^3^ versus 5.65 mm^3^; p < 0.0001, Fig. 2D) were also significantly smaller than in WT mice. There were no significant differences in brain volumes, hippocampal volume, or DG volume in mutant or WT mice undergoing Morris water maze compared to the forced swim test.

### Acquisition and long-term memory in the MWM

To understand the influence of dentate neurogenesis on hippocampal-dependent spatial memory, we examined the cyclin D2^−/−^ mice using a tailored battery of behavioral tests modified with long-term memory probes. Each behavioral task was validated for the ability of WT C57BL6/J mice to retain the information required for long-term intervals in independent cohorts of mice (Supplemental Figs. 1, 2 for MWM and pattern separation; Supplemental Table 1). We also ruled out impaired locomotor activity of cyclin D2^−/−^ mice in an open field test. No genotype differences were observed in total locomotor activity, horizontal, and vertical rearing, or time spent in the center of the apparatus (Supplemental Fig. 3).

**Table 1:** Statistical data of Morris water maze, easy location touchscreen discrimination task and pattern separation touchscreen task.

**Figure**	**Test**	**Sample Size**	**Metric**	**Time Point**	**Statistical Test**	**Statistic**	**p value**	**Posthoc test**	**p value**
3, A	Acquisition of Morris Water Maze	WT N = 20, Cyclin D2^−/−^ N = 27	Latency to platform (s)	Day 1	Two Way Repeated Measures ANOVA	Day F (6,270) = 27.55 Genotype F (1,45) = 7.341 Interaction F (6,270) = 1.441	p < 0.0001^*^ p = 0.0095^*^ p = 0.1991	Bonferroni WT vs. Cyclin D2^−/−^	p = 0.9930
				Day 2					p = 0.9029
				Day 3					p = 0.5290
				Day 4					p = 0.9779
				Day 5					p = 0.0740^†^
				Day 6					p = 0.0115^*^
				Day 7					p = 0.0896^†^
	**Test**	**Group**	**Day 1 vs. 2**	**Day 1 vs. 3**	**Day 1 vs. 4**	**Day 1 vs. 5**	**Day 1 vs. 6**	**Day 1 vs. 7**	
3, A	Posthoc within genotype, between days	WT	p = 0.0753^†^	p = 0.0006^*^	p < 0.0001^*^	p < 0.0001^*^	p < 0.0001^*^	p < 0.0001^*^	
		Cyclin D2^−/−^	p = 0.1100	p = 0.0063^*^	p < 0.0001^*^	p < 0.0001^*^	p < 0.0001^*^	p < 0.0001^*^	
**Figure**	**Metric**	**Group**	**One Way ANOVA**	**p value**	**Bonferroni Post Hoc**				
			**F Statistic**		**T vs. L**	**T vs. R**	**T vs. O**		
3, B	3-hr Probe, Time in Quadrant	WT	F (3, 76) = 19.83	p < 0.0001^*^	p < 0.0001^*^	p < 0.0001^*^	p < 0.0001^*^		
		Cyclin D2^−/−^	F (3, 104) = 6.481	p = 0.0005^*^	p = 0.0013^*^	p = 0.0023^*^	p = 0.5898		
3, C	24-hr Probe, Time in Quadrant	WT	F (3, 76) = 18.64	p < 0.0001^*^	p < 0.0001^*^	p = 0.0002^*^	p < 0.0001^*^		
		Cyclin D2^−/−^	F (3, 104) = 3.033	p = 0.0326^*^	p = 0.0371^*^	p = 0.1125	p = 0.9715		
3, D	1-week Probe, Time in Quadrant	WT	F (3, 72) = 2.759	p = 0.0484^*^	p = 0.0463^*^	p = 0.1834	p = 0.0473^*^		
		Cyclin D2^−/−^	F (3, 104) = 1.713	p = 0.1689	p > 0.9999	p = 1146	p > 0.9999		
3, E	2-week Probe, Time in Quadrant	WT	F (3, 76) = 16.42	p < 0.0001^*^	p < 0.0001^*^	p = 0.0010^*^	p < 0.0001^*^		
		Cyclin D2^−/−^	F (3, 104) = 0.838	p = 0.4760	p > 0.9999	p = 0.4394	p > 0.9999		
3, F	4-week Probe, Time in Quadrant	WT	F (3, 76) = 10.23	p < 0.0001^*^	p = 0.0006^*^	p = 0.0003^*^	p < 0.0001^*^		
		Cyclin D2^−/−^	F (3, 104) = 2.354	p = 0.0763^†^	p > 0.9999	p > 0.9999	p = 0.1100		
**Two-way RM ANOVA**					**Post hoc comparison WT vs Cyclin D2**^**−/−**^ **Quadrant P value**				
**Figure**	**Group**	**Test**	**F Statistic**	**P value**	**T**	**L**	**R**	**O**	
3, B	MWM 3 hours	Quadrant	F (3, 135) = 15.93	p < 0.0001^*^	p = 0.0108^*^	p > 0.9999	p > 0.9999	p = 0.0045^*^	
		Genotype	F (1, 45) = 0.05426	p = 0.8169					
		Interaction	F (3, 135) = 5.179	p = 0.002^*^					
3, C	MWM 24 hours	Quadrant	F (3, 135) = 11.11	p < 0.0001^*^	p = 0.0087^*^	p > 0.9999	p > 0.9999	p = 0.0246^*^	
		Genotype	F (1, 45) = 0.7365	p = 0.3953					
		Interaction	F (3, 135) = 4.805	p = 0.0033^*^					
3, D	MWM 1 week	Quadrant	F (2.372, 104.4) = 2.404	p = 0.0857	p > 0.9999	p > 0.9999	p > 0.9999	p = 0.9106	
		Genotype	F (1, 44) = 0.07561	p = 0.7846					
		Interaction	F (3, 132) = 1.083	p = 0.3585					
3, E	MWM 2 weeks	Quadrant	F (2.310, 103.9) = 7.518	p = 0.0005^*^	p = 0.0133^*^	p = 0.041^*^	p > 0.9999	p = 0.0664	
		Genotype	F (1, 45) = 2.391	p = 0.1290					
		Interaction	F (3, 135) = 6.068	p = 0.0007^*^					
3, F	MWM 4 weeks	Quadrant	F (2.546, 114.6) = 2.285	p = 0.0927	p = 0.0092^*^	p > 0.9999	p > 0.9999	p = 0.0027^*^	
		Genotype	F (1, 45) = 0.1424	p = 0.7077					
		Interaction	F (3, 135) = 5.830	p = 0.0009^*^					
**Figure**	**Test**	**Sample Size**	**Metric**	**Statistical Test**	**Statistic**	**P value**			
5, C	Easy Location Discrimination Touchscreen Task	WT N = 10 Cyclin D2^−/−^ N = 11	% of Animals Completed	Gehan-Breslow-Wilcoxon Test	X = 0.7760	p = 0.3784			
5, D			Sessions to Criteria	Unpaired Two-Tailed T-Test	T (19) = 0.9885	p = 0.3353			
5, E			# of correct reversals, 1-week probe	Unpaired Two-Tailed T-Test	T (18) = 0.2535	p = 0.8028			
5, E			# of correct reversals 2-week probe	Unpaired Two-Tailed T-Test	T (18) = 0.5569	p = 0.5778			
5, E			# of correct reversals 4-week probe	Unpaired Two-Tailed T-Test	T (18) = 1.789	p = 0.0905			
5, E			# of correct reversals 8-week probe	Unpaired Two-Tailed T-Test	T (18) = 0.03204	p = 0.9748			
**Figure**	**Test**	**Sample Size**	**Metric**	**Statistical Test**	**Statistic**	**P value**			
6, C	Pattern Separation Touchscreen Task	WT N = 10 Cyclin D2^−/−^ N = 12	% of Animals Completed	Gehan-Breslow-Wilcoxon Test	X = 0.6075	p = 0.4357			
6, D			Sessions to Criteria	Unpaired Two-Tailed T-Test	T (20) = 0.4302	p = 0.6717			
6, E			# of correct reversals, 1-week probe	Unpaired Two-Tailed T-Test	T (20) = 1.528	p = 0.1422			
6, E			# of correct reversals 2-week probe	Unpaired Two-Tailed T-Test	T (20) = 2.225	p = 0.0378^*^			
6, E			# of correct reversals 4-week probe	Unpaired Two-Tailed T-Test	T (20) = 2.227	p = 0.0376^*^

**Figure 3:**
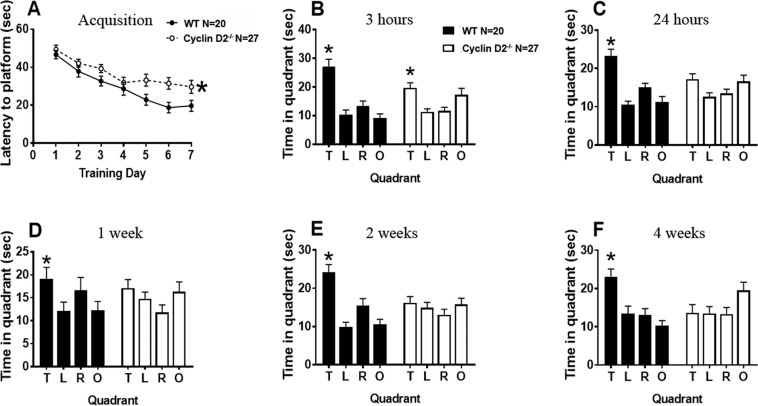
Cyclin D2^−/−^ mice show normal Morris water maze acquisition and short-term memory but deficits in long-term memory. (**A**), Both WT and cyclin D2^−/−^ mice display normal performance during acquisition as latency to platform significantly decreases across training days. There are significant genotype differences (Repeated Measures Two-Way ANOVA: F_(1,45)_ = 7.341, p < 0.05*). (**B**), Both WT and cyclin D2^−/−^ mice exhibit intact short-term memory at the 3-hour probe and spent more time in the target quadrant compared to the others during the 60 s probe trial. (**C**), At the 24-hour probe, WT mice show intact memory, but cyclin D2^−/−^ mice do not. (**D–F)**, Cyclin D2^−/−^ have impaired long-term memory and do not spend more time in the target quadrant compared to remaining quadrants at the 1-week, 2-week, and 4-week probes. *p < 0.05 indicates main effect of genotype in repeated measures two-way ANOVA (A), p < 0.05 indicates significant difference between target quadrant and others (B-F). T = Target, L = Left, R = Right, O = Opposite. Sample size: WT N = 20, CyclinD2^−/−^ N = 27, mixed sex. For statistical data see Table 1.

**Figure 4:**
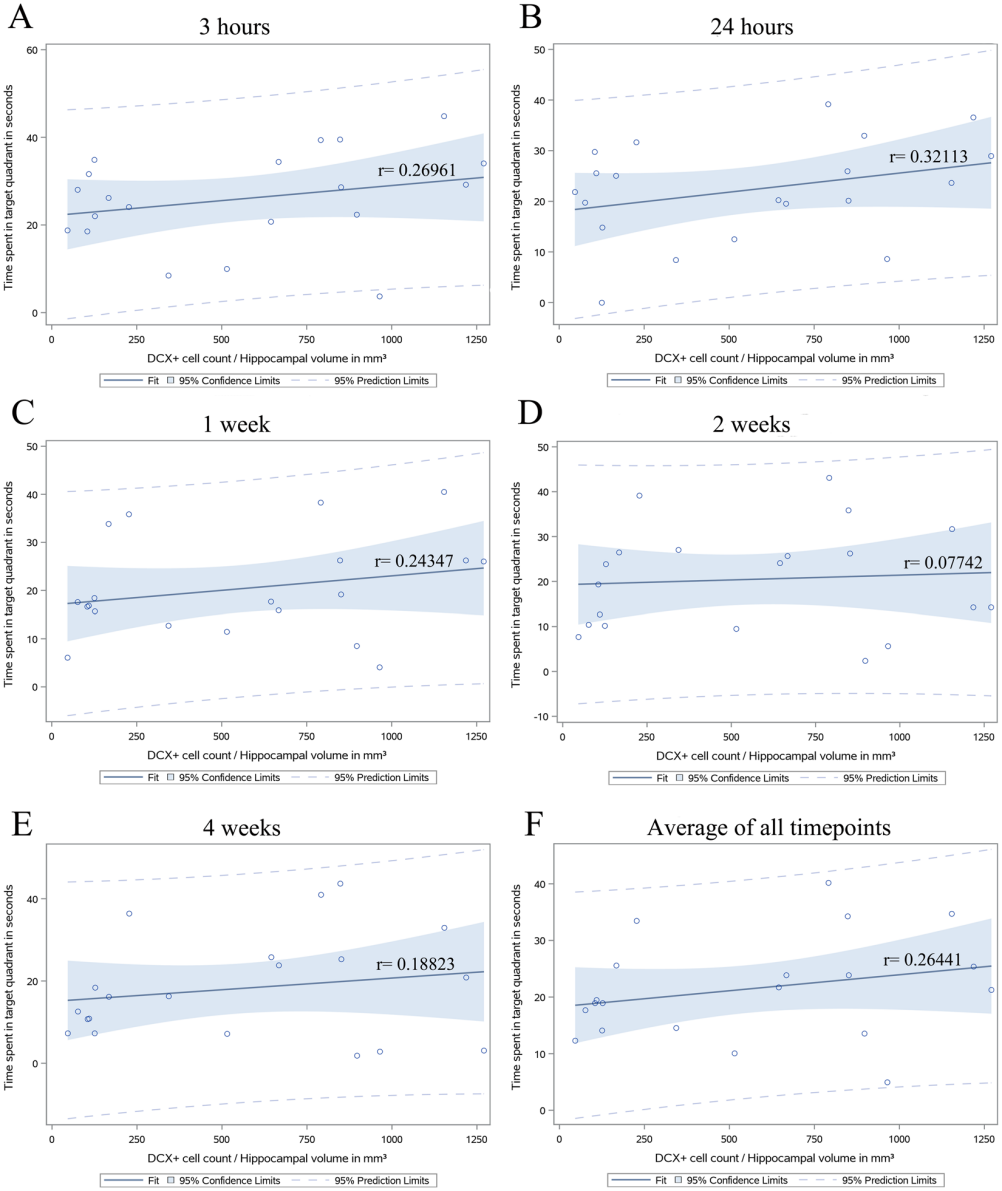
Dentate neurogenesis adjusted for differences in hippocampal volume moderately correlates with time spent in the target quadrant 24 hours after Morris water maze acquisition learning. (**A**) A weak correlation was found between the number of DCX^+^ cells normalized to hippocampal volume and time spent in target quadrant 3 hours after acquisition. (**B**) A moderate correlation (r = 0.32113, F_(1,18)_ = 2.07, p = 0.1674) between DCX^+^ cell count adjusted for hippocampal volume and time spent in target quadrant was seen 24 hours after acquisition. (**C–F**) the correlation between DCX^+^ cell count adjusted for hippocampal volume was weaker at later time points and averaged over all timepoints.

During the acquisition phase of the MWM, WT mice performed significantly better compared to cyclin D2^−/−^ in latency to reach the platform (F_(1,45)_ = 7.341, p < 0.05), but both WT and cyclin D2^−/−^ mice were able to learn the location of the platform in a 7-day acquisition task (Fig. 3A), and cyclin D2^−/−^ mice had significantly slower swim speeds (Supplemental Fig. 4). Both WT and cyclin D2^−/−^ mice had intact spatial memory 3 hours after initial MWM testing (Fig. 3B). Only cyclin D2^−/−^ showed attenuated spatial memory after 24 hours (Figs. 3C), 1 week (Figs. 3D), 2 weeks (Figs. 3E) and 4 weeks (Fig. 3F), while WT mice had intact long-term spatial memory at all tested timepoints (Table 1). To enhance the rigor of the study, a separate cohort of animals was tested, and results were reproduced (Supplemental Fig. 5). Probe trial differences between genotypes were also reproduced when assessing distance traveled per quadrant instead of time spent in each quadrant (Supplemental Fig. 6). In addition, measures recorded on probe trials are insensitive to swimming speed^29^. Finally, both sexes were tested, and no sex differences were observed. Therefore, we concluded that cyclin D2^−/−^ mice had robust deficits in the establishment of long-term memory starting at 24 hours following acquisition training.

**Figure 5:**
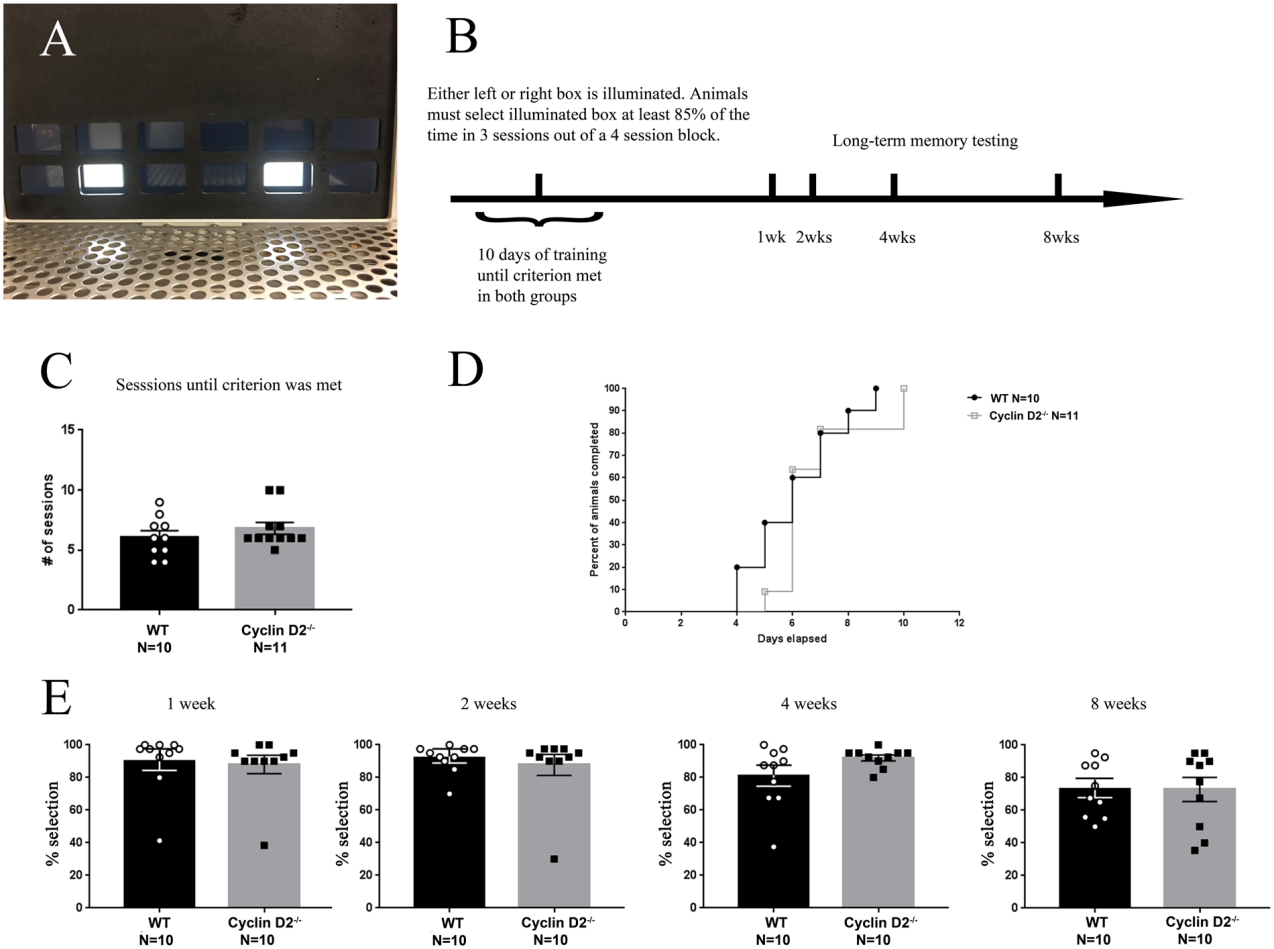
Cyclin D2^−/−^ mice are able to learn a visual discrimination task. (**A**) The touchscreen chamber for the visual discrimination task was set up to contain two lit boxes which were separated by two unlit boxes. (**B)** Graphical outline of the experimental paradigm for the visual discrimination task. (**C**) No genotype difference was seen in the number of sessions required to reach criterion of 80% selection for two consecutive days, indicating normal acquisition of the task. (**D**) Days to criterion, representing the proportion of individual subjects that completed acquisition at each training day (survival curve). Cyclin D2^−/−^ mice were able to acquire the visual discrimination task equally well as WT mice. (**E**) Memory was tested at 1, 2, 4, and 8 weeks after the completion of acquisition. There were no genotype differences at any time point.

**Figure 6:**
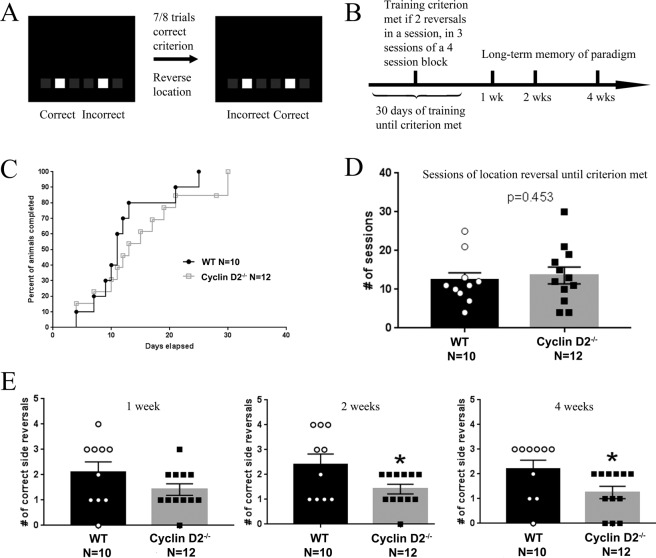
Cyclin D2^−/−^ mice displayed deficits in long-term but not short-term memory in an intermediate pattern separation task. (**A**) Mice were tested in a touchscreen chamber with the same design as in the two-choice discrimination task (Fig. 5). (**B)** Graphical plot of experimental paradigm for intermediate pattern separation task. (**C–D)** No differences were observed between genotypes in number of days to reach criterion of 2 reversals in a session, in 3 sessions of a 4-session block, indicating intact acquisition of the task. (**E)** In a one-session probe trial one week later, there was no genotype difference in the number of correct side reversals performed. At the two-week probe session, cyclin D2^−/−^ mice performed fewer correct side reversals, indicating a long-term memory, spatial pattern deficit (two-tailed unpaired t-test, p < 0.05). At the four-week probe session, cyclin D2^−/−^ again displayed fewer number of correct side reversals (two-tailed unpaired t-test p < 0.05). * p < 0.05 Sample size: WT N = 10, cyclin D2^−/−^ N = 12.

### Moderate correlation between dentate neurogenesis and long-term spatial memory at 24 hours

Next, we asked whether there is a correlation between dentate neurogenesis and recall of long-term spatial memory in the MWM. We chose doublecortin expression as a marker of dentate neurogenesis given that it reflects a population of cells committed towards neuronal lineage. Since MRI brain showed significant differences in the neuroanatomy of cyclin D2^−/−^ and WT brains, we adjusted the correlation of dentate neurogenesis to spatial memory for differences in hippocampal volumes within each animal. We verified with swim stress control experiments that physical exercise did not lead to significant changes in brain volumes.

Three hours after acquisition, the correlation between MWM performance and dentate neurogenesis adjusted for hippocampal volume was <0.3 (r = 0.26961, Fig. 4A). We found a moderate correlation^30^ between dentate neurogenesis adjusted for hippocampal volume and spatial memory 24 hours after acquisition (r = 0.32113, Fig. 4B). At later timepoints, the correlation was weaker including at 1 week (r = 0.24347, Figs. 4C), 2 weeks (r = 0.07742, Fig. 4D), and 4 weeks (r = 0.18823, Fig. 4E). The correlation of the average of all timepoints was also weaker than the correlation at 24 hours after acquisition (r = 0.26441, Fig. 4F). We repeated this analysis, adjusting for total brain volume rather than hippocampal volume and achieved similar results (Supplemental Figure 7) as there was a moderate correlation at the 24-hour time point and weaker correlations at all other time points.

### Cyclin D2^−/−^ mice have intact intermediate pattern separation

To evaluate the suitability of cyclin D2^−/−^ mice for a pattern separation task, we first tested whether cyclin D2^−/−^ mice could remember the location of correct visual stimuli in a pairwise touchscreen visual discrimination paradigm (Supplemental Figure 8). In this non-hippocampal, non-spatial visual discrimination task, cyclin D2^−/−^ animals performed similarly to WT, requiring a similar number of sessions to reach criterion to acquire the task (cyclin D2^−/−^: 29.78 ± 0.68 sessions, WT: 29.50 ± 0.66 sessions). Next, we tested if mice of both genotypes could perform an easy, non-spatial choice discrimination task in the touchscreen apparatus (Fig. 5A). Animals underwent 10 days of training until the criterion of a response level of 85% in 3 sessions out of a 4-sessions block was met. Animals underwent probe sessions at several later time points after acquisition to test their long-term memory of the task (Fig. 5B). Cyclin D2^−/−^ mice did not show a delay in acquisition learning compared to WT mice (t_19_ = 0.9885, p > 0.05, Fig. 5C,D). Cyclin D2^−/−^ mice also did not show deficits in long-term memory of this easy task compared to WT at all later timepoints probed (Fig. 5E; 1 week: t_19_ = 0.3353, p > 0.05, 2 weeks: t_18_ = 0.5778, p > 0.05, 4-weeks: t_18_ = 0.0905, p > 0.05, 8 weeks: t_18_ = 0.9748, p > 0.05).

After verifying that cyclin D2^−/−^ mice had intact visual location memory, we tested them in an intermediate pattern separation paradigm. Our goal was to test whether dentate neurogenesis had a stronger influence on intermediate pattern separation or on long-term memory formation. Animals were tested in a touchscreen chamber with two illuminated squares, and stimuli were reversed after >85% correct selections until the criterion of 2 reversals in a single session, in 3 sessions of a 4-session block, was met (Fig. 6A,B). Subsequently, we tested for long-term memory of the reversal paradigm 1 week, 2 weeks, and 4 weeks after acquisition (Fig. 6B). Cyclin D2^−/−^ mice were not inferior to WT mice in reaching criterion after 30 days of acquisition training (Fig. 6C). WT mice required on average 12.3 ± 1.98 sessions, and cyclin D2^−/−^ required 13.58 ± 2.16 sessions until criterion was reached (p = 0.453, Fig. 6D). Thus, cyclin D2^−/−^ mice showed no significant deficits in intermediate pattern separation compared to WT mice. However, cyclin D2^−/−^ animals performed worse than WT animals 1 week after acquisition (t_20_ = 1.528, p = 0.1422), and significantly worse 2 weeks (t_20_ = 2.225, p < 0.04) and 4 weeks (t_20_ = 2.227, p < 0.04) after acquisition in their long-term memory of the pattern separation task (Fig. 6E). Again, we did not identify any sex differences in any metric studied. In summary, we did not detect deficits in cyclin D2^−/−^ animals in acquisition of an intermediate pattern separation task, but only in establishing a long-term memory formation of this task.

## Discussion

In summary, using a genetic model of attenuated dentate neurogenesis, we found profound, reproducible, long-term memory deficits on two behavioral assays with no impairment in acquisition or short-term memory, and no impairment in intermediate pattern separation. Additionally, we found a moderate correlation between reduced dentate neurogenesis and long-term memory impairment in MWM at 24 hours adjusted for differences in hippocampal volumes.

In the last decade, several reviews have focused on the importance of dentate neurogenesis for pattern separation^31–33^. However, the pattern separation model has been challenged by several computational models that have shown a decrease in pattern separation with neurogenesis^34–36^. While fMRI studies have shown a role for the dentate gyrus and CA3 region in pattern separation in humans, investigators could not confidently isolate the dentate gyrus from CA3 with imaging^37^. Therefore, it is also possible that pattern separation/completion is predominantly performed by CA3, and integrated dentate progenitor cells serve as amplifiers to rate the importance of an episodic memory information and facilitate its transfer into long-term memory based on importance. We therefore investigated the hypothesis that the main function of dentate neurogenesis is long-term memory formation over the more prominently researched function, pattern separation.

In addition to using standard behavioral classics, such as the water maze, we designed novel touchscreen experiments that directly compared intermediate pattern separation to long-term memory formation. There is no uniform definition for a low, intermediate or high pattern separation setting of the touchscreen apparatus in the current literature. The original publication by McTighe *et al*. utilized a distance of three unlighted boxes for intermediate pattern separation and one unlighted box for minimum pattern separation^38^, likely optimizing distances due to the study goal of examining hippocampal lesions in rats, a larger rodent. Clelland *et al*. chose a distance of three unlighted boxes for high pattern separation and one unlighted box for low pattern separation^1^, optimizing for mice. The work by Creer *et al*. used two unlighted boxes as an intermediate pattern separation distance for training of their pattern separation task, however the probe trials to delineate the dependent variables of aging and exercise used 4 unlighted boxes as stimulus distance for high pattern separation and no unlighted box for low pattern separation^2^. We used an intermediate pattern separation task with spatial measurements identical to Creer *et al*. for training the pattern separation task as established above. Since we wanted to directly compare long-term memory to pattern separation in our paradigm, we had to avoid designing a pattern separation task so challenging that even WT mice could not perform the long-term recall, as we would expect with a low pattern separation design. We therefore used an intermediate pattern separation task, and we did not find significant differences between cyclin D2^−/−^ animals and controls suggesting that dentate neurogenesis is not essential for intermediate pattern separation in our animal model.

However, when we tested intermediate pattern separation and long-term memory with probes in touchscreen, we found a significant difference between cyclin D2^−/−^ and WT. One week after testing, WT animals performed better than cyclin D2^−/−^, although these data only trended to statistical significance. There was a significant reduction in recall of the intermediate pattern separation paradigm 2 weeks and 4 weeks after acquisition learning. Since acquisition of intermediate pattern separation learning was unchanged between cyclin D2^−/−^ and WT but long-term memory was impaired, cyclin D2^−/−^ mice with attenuated neurogenesis may have a more prominent deficit in long-term memory formation than in intermediate pattern separation. We do not claim that dentate neurogenesis has no role for pattern separation as we did not test low pattern separation designs, and as Clelland *et al*. convincingly illustrated the role of dentate gyrus neurogenesis in pattern separation^1^. Therefore, our results support the rich literature from the Saksida-Bussey groups on touchscreen pattern separation experiments. However, based on our results we propose that pattern separation may be a secondary deficit, as our data illustrate that long-term memory of intermediate pattern separation outweighed acquisition of intermediate pattern separation, and as there was a profound longitudinal long-term memory deficit in the MWM in our model.

In contrast to previous findings, we did not find deficits of cyclin D2^−/−^ mice with decreased dentate neurogenesis in flexible learning^6^ or reversal learning^7^. Our results are in agreement with Jaholkowski *et al*. who have also shown intact reversal learning of cyclin D2^−/−^ mice in the IntelliCage system^11^. Our results are in disagreement with Filipkowski and Kascmarek’s extensive behavioral investigations on cyclin D2^−/−^ mice, which suggest that there are no major deficits in long-term learning and memory in cyclin D2^−/−^ mice^39^. They detected slight memory deficits one week after acquisition in the MWM^40^. However, at all long-term memory time points tested in their study (24 hours, 1 week, 2 weeks, and 3 weeks after acquisition), cyclin D2^−/−^ mice spent more time in the target quadrant than would be predicted by chance, indicating they had intact long-term memory. Significant differences were only observed relative to the performance of WT animals 1 week after acquisition. In our study, we observed that cyclin D2^−/−^ mice spent no significant time in the target quadrant at 24 hours or later long-term memory timepoints and therefore had persistent impairment in long-term memory across the experimental time course. A possible explanation for the discrepant results between our study and Filipkowski *et al*. is the genetic background of our model. We received cyclin D2^−/−^ mice on a mixed C57BL/6 and Sv129 background. We rederived cyclin D2^−/−^ mice with C57BL/6J female donors so mice may have had a different genetic background than the cyclin D2^−/−^ mice used in previous studies. It is well established that the genetic background may influence the behavioral phenotype^41^.

Our study is in agreement with other investigators who reported long-term memory deficits with impaired neurogenesis after 1 week or later time points^9,20,25^. However, results on neurogenesis and long-term memory have been discrepant between investigators. Snyder *et al*., for example, found no memory deficits at 1 week but did at 2 weeks and 4 weeks after acquisition in the MWM in animals with reduced neurogenesis following radiation^10^. Imayoshi *et al*. did not detect memory deficits 24 hours after Morris water maze training in animals with reduced neurogenesis^20^. In contrast, we observed a memory deficit in MWM that was strongly associated with the cyclin D2^−/−^ genotype across all time points beyond 3 hours. Our data suggest that the cyclin D2^−/−^ genotype is important for long-term memory formation, and behavioral effects can be seen as early as 24 hours after training. There is evidence that neurogenesis is enhanced by LTP^24^, which suggests the possibility that adult dentate neurogenesis is the extended arm of LTP to ‘lock-in’ episodic memories until they are consolidated outside the hippocampus. Altogether, we believe our data is strong because it links the cyclin D2^−/−^ genotype to long-term memory in robust longitudinal findings that were reproduced in a separate cohort. We do not think that the long-term memory performance of WT mice in the MWM was a reconsolidation of contextual memory in the setting of longitudinal testing since we would have expected an increase in probe trial scores in WT mice as the number of contextual reinforcements increases over time, but this was not the case.

The downside of our model is that cyclin D2^−/−^ mice have other neuroanatomical differences compared to WT, besides decreased neurogenesis. This is a limitation of many gene-targeted models because the encoded protein is missing throughout development which may affect multiple endpoints in the adult brain. We were mindful of this limitation of our genetic model and made an effort to control for these differences by adjusting normalized neurogenesis counts for changes in hippocampal volume and by demonstrating that the cyclin D2^−/−^ mice were able to learn all of the tasks. The advantage of our model is that it does not involve an external manipulation to decrease neurogenesis. Even though other investigators have not seen neuro-inflammation with their ablative techniques^25^, subtle inflammatory changes induced by neuro-ablation may be difficult to capture and could most certainly have an impact on behavioral outcomes. Microglial activation detected by changes in soma volume has been linked to impaired long-term recognition memory^42^, and neuroinflammation is hypothesized to contribute to behavioral phenotypes of cognitive impairment^43,44^.

In conclusion, we have demonstrated that cyclin D2^−/−^ mice with attenuated dentate gyrus neurogenesis have robust deficits in long-term memory formation across two behavioral tasks, and that these deficits play a more important role in the long-term memory formation of an intermediate pattern separation task than in the acquisition learning of the same task. Therefore, our data support a model in which the main deficit of cyclin D2^−/−^ mice with decreased dentate gyrus neurogenesis is long-term memory formation. Our conclusions are limited by the presence of other neuroanatomical changes in cyclin D2^−/−^ mice such as decreased olfactory bulb, hippocampus, cerebellum, and cortex, as we have shown with MRI and adjusted for in this study. Further experimental work is necessary to validate the importance of dentate neurogenesis for long-term memory formation such as through enhancement of dentate gyrus neurogenesis in the cyclin D2^−/−^ mouse model.

## Methods

### Cyclin D2^−/−^ mice

All experimental protocols involving animals were approved by the UC Davis Institutional Animal Care and Use Committee (IACUC). One male and female cyclin D2^+/−^ mouse from a mixed C57BL/6 and Sv129 background were received from Dr. Peter Sicinski at Dana-Farber Cancer Institute (Boston, MA, USA). The mice were bred to C57BL/6J mice upon arrival to generate additional males for rederivation of the genetic line into the barrier facility. *In vitro* fertilization was performed using C57BL/6J female donors and two cyclin D2^+/−^ males. We transferred 52 embryos into two female recipients (26 embryos per female), and 5 cyclin D2^+/−^ offspring were produced (1 female, 4 males). Multiple breeder pairs were generated from the initial animals at approximately 2-3 months of age. Pairs were kept together, and subsequent offspring were genotyped using Transnetyx (Cordova, TN, USA) with primers for cyclin D2^+/+^ (WT)  and cyclin D2^−/−^ shown in Table 2. Dams produced up to 6 litters before being retired. If a pair did not produce offspring following 2 months of mating, they were given new partners or euthanized if too old (over 12 months of age). We used age- and sex-matched littermates as controls for this behavioral genetic study as previously described^45,46^.

**Table 2:** Primer sequences used for genotyping of WT and cyclin D2^−/−^.

Genotype	Forward Primer	Reporter 1	Reverse Primer
WT	AAGGTTCTGCAGCTCTCTTTTACA	AAGGTGTGCAAACCAC	GCAGGCCCAGGCAATG
Cyclin D2^−/−^	GCCCTCGAGCTCTGTACATG	TCCGCGGTCGGTAGTT	TGGGTTTTTTGCAGGAAGTTAGGAT

### Experimental design

All animals were cared for in accordance with the guidelines and procedures set by the National Institutes of Health and the Institutional Animal Care and Use Committee at the University of California Davis (Sacramento, CA, USA). Animals were housed 2-4 per cage in a temperature and humidity-controlled vivarium with a 12-hour light/dark cycle and provided food and water ad libitum. Cohort 1 was tested in the Morris water maze and open field assays starting at 6 weeks of age. In the Morris water maze, there were four subgroups: WT (water maze trained and probed; N = 20; 14 female and 6 male); WT (swim stress control; N = 15; 8 female and 7 males); cyclin D2^−/−^ (swim stress control; N = 15; 6 female and 9 male); cyclin D2^−/−^ (water maze trained and probed; N = 27; 13 female and 14 male). Swim stress was used as a control to ensure that physical exercise did not lead to changes in brain volumes on magnetic resonance (MR) imaging. A second cohort (Cohort 2) was later tested to replicate MWM phenotypes (WT N = 14; cyclin D2^−/−^ N = 11). Approximately equal numbers of each sex were tested for cohort 2.

**Table 3:** Antibodies used to detect neurogenic markers.

RRID	Antibody	Manufacturer	Catalog number	Lot	Dilution
AB_443209	Ki67	Abcam	15580	GR3198167–1	1:750
AB_1586992	DCX	Millipore	AB2253	3059059	1:500
AB_2576217	Alexa Fluor 488	Invitrogen	A11034	1812166	1:500
AB_2534119	Alexa Fluor 568	Invitrogen	A11075	1885925	1:1000

### Morris water maze

Spatial learning was assessed using a Morris water maze (MWM) assay as previously published by our laboratory^47,48^ with additional long-term memory probes that were validated within this study (Supplemental Fig. 1, Supplemental Table 1). The apparatus was a circular pool (120 cm diameter) filled 45 cm deep with tap water made opaque with the addition of non-toxic white paint (Crayola, Easton, PA). Distal room cues included black and white cardboard patterns on the walls located approximately 1 m from the edge of the pool. Water temperature was maintained at 23 °C. Trials were digitally recorded and analyzed using EthoVision XT software (version 9.0, Noldus Information Technologies, Leesburg, VA, USA). Acquisition training consisted of 4 trials per day for 7 consecutive days. The hidden platform remained in the same quadrant for all trials during acquisition training for a given mouse but varied across subject mice, randomized within genotype. Each training trial began by lowering the mouse into the water close to the pool edge, in a quadrant that was either right of, left of, or opposite to the target quadrant containing the hidden platform. The start location for each trial was alternated in a semi-random order for each subject. Trials consisted of a maximum time of 60 seconds for mice to reach the platform. Mice were allowed 15 seconds on the platform before removal. If a mouse failed to reach the platform, it was guided to the platform by the experimenter using a wire cage lid. After the completion of each trial, mice were placed in a clean cage lined with paper towels and allowed to rest under a warm infrared lamp. During probe trials, the hidden platform was removed, and mice were tested again for a maximum time of 60 seconds to determine their spatial learning and memory by using distal environmental cues. Probe trials were conducted 3 hours, 24 hours, 1 week, 2 weeks and 4 weeks after the final acquisition trial. These long-term memory times were validated in WT C57BL6/J mice for retention of platform location via probe trials out to 2 months post training (N = 16; Supplemental Fig. 1C). Parameters assessed during acquisition training and probe trials were latency to the platform (in seconds), time spent and distance traveled in each quadrant, distance traveled to platform, and swim speed (cm/second).

### Touchscreen apparatus

Learning and memory was assessed using the automated Bussey-Saksida touchscreen apparatus for mice (Campden Instruments Ltd./Lafayette Instruments, Lafayette, IL, USA), using procedures based on previously described methods^49–51^ that were modified to test our current hypothesis. Prior to pre-training, mice were weighed and placed on a restricted diet of 2-4 g of standard rodent chow per mouse per day to achieve a 15% weight loss. Body weight was monitored throughout the experiment and mice were maintained at 85% or more of free-feeding body weight for the duration of the experiment. The reinforcer was 20 µL of a palatable liquid nutritional supplement (Strawberry Ensure Plus, Abbott, IL, USA) diluted to 50% with water. Each session was conducted under overhead lighting (~60 lux). A tone cue was used to signal the delivery of the reinforcer during pre-training and acquisition. All data were automatically collected by the touchscreen system.

### Non-spatial choice discrimination task

To test non-spatial choice discrimination in which there were two different locations illuminated, one location (left or right) was illuminated. This location was always reinforced, without any reversal or spatial component. We created this easy, same location, same side procedure that was modified from previously described methods^38^. During pre-training, a mask over the touchscreen with 6 windows was used. Windows 2 and 5 were designated as right or left. If right was illuminated, it was always reinforced and vice versa for left. We termed this an ‘easy choice’ task with no reversal component and tested 21 animals (WT N = 10, Cyclin D2^−/−^ N = 11).

Pre-training consisted of 4 stages. Stage 1 consisted of 2 days (20 minutes on the first day, 40 minutes on the second day) of habituation to the operant chamber and freely available liquid diet under overhead lighting with nothing lit on the screen. Stage 2 was a single session during which one white square was illuminated randomly in windows 1 through 6. This stimulus remained for 30-seconds then disappeared. A food reward was then given in an illuminated tray and a tone was played. Subjects were given 3 times the normal reward for touching the stimulus square while illuminated with no punishment for incorrect touches and a 30-second inter-trial interval before reward delivery. The criterion to advance to the next stage was completion of 30 trials in 60 minutes. During Stage 3, a single white square was presented randomly in windows 1 through 6 and remained lit until it was touched. This was accompanied by the delivery of a food reward in the illuminated food tray and an audible tone. For incorrect touches on the blank part of the screen, there was no response. Subjects were required to complete 30 trials per daily session for 2 consecutive days to advance to the final stage. Finally, in Stage 4, mice were trained that touching an unlit window on the screen was discouraged resulting in a 5-second time-out during which the overhead light was turned on. Subjects needed to achieve completion of 23 out of 30 trials in 30 minutes on 2 consecutive days to meet the criterion for advancement to the experimental probe session. Pre-training sessions were limited to 60 minutes. Mice that successfully completed all pre-training stages were used in the experimental probe. After pre-training, subjects were trained to nose-poke 1 of the 2 lit windows in order to be rewarded. Subjects began with either the right or left side assigned as illuminated and reinforced, and the task was counterbalanced across subjects and sexes within each genotype. When subjects touched the illuminated side, they were given a reward to reinforce the choice.

### Spatial location discrimination task

To test hippocampal pattern separation and memory, our location discrimination task used a procedure modified from previously described methods^2,38^. During pre-training, a mask with 6 windows was used. Windows 2 and 5 were designated as right or left. Pre-training was conducted exactly as described above for the non-spatial task. Only mice that successfully completed all pre-training stages were used in the experiment. After pre-training, subjects were trained to nose-poke a lit window in order to be rewarded. Subjects began with either the right or left side assigned as correct and the task was counterbalanced across subjects and sexes within each genotype. When subjects touched the correct side, they were rewarded. When mice touched the incorrect side, the house light was illuminated for a 5-second time-out. If 7 out of 8 stimuli presentations were correctly pressed by subjects, the correct side was then reversed. Subjects had 60-minute daily sessions to perform as many reversals as possible. Criterion for acquisition is 2 reversals in a single session across 3 sessions within a 4-session block. The number of days to reach criterion was analyzed between genotypes. After acquisition of the task, a novel approach to testing long term spatial memory was employed by conducting probes longitudinally. After the completion of the acquisition phase, probe sessions were conducted at 1, 2- and 4-weeks post-acquisition on an individual basis. Animals progressed through the task and probe sessions as individuals, not as a group, thus every animal was tested with consistent inter-probe intervals. Animals were given a single 60-minute session to perform as many reversals as possible. The number of correct reversals made during the probe session were recorded and analyzed (WT N = 10, Cyclin D2^−/−^ N = 12).

### Pairwise Discrimination

Pairwise discrimination was conducted as previously described^49^. During pre-training, a mask with 2 windows was used. In Stages 3-4, random images were used, and the images were not repeated during the acquisition of the paradigm. After pre-training, subjects were trained to discriminate between 2 novel images: a spider and an airplane, which are presented in a spatially pseudo-randomized manner. Each 45-minute session consisted of an unlimited number of trials separated by 15-second inter-trial intervals (ITI). Designation of the correct and incorrect images was counterbalanced across mice within each genotype. Correct responses were rewarded. Each incorrect response was followed by a correction trial in which the images were presented in an identical manner to the previous trial, until a correct response was made. Criterion for completion was completing at least 30 trials, at an accuracy of 80% or higher, on 2 consecutive days. Sessions to criterion and proportion of subjects per genotype completing criterion per day were assessed and compared between genotypes (WT N = 9; Cyclin D2^−/−^ N = 10).

### Open Field

General exploratory locomotion in a novel open field environment was assayed (WT N = 15; Cyclin D2^−/−^ N = 12) as previously described^49,52^. Open field activity was considered an essential control for effects on physical activity, e.g. sedation or hyperactivity, which could confound the interpretation of results from the water maze. The testing room was illuminated at ~30 lux.

### Statistical analyses for behavioral tasks

All statistics and graphing for behavioral experiments were done with GraphPad Prism (Version 7, GraphPad Software, San Diego, CA, USA). Morris water maze acquisition training was tested using a two-way repeated measures ANOVA with factors of training day and genotype. Morris water maze probe trials were analyzed by one-way ANOVA with *post hoc* Bonferroni analysis contingent upon significant main ANOVA effect as is standard for this task^29^. We verified that the assumptions of normality and constant variance in MWM are true with QQ plots, residuals plot, and D’Agostino and Pearson test in validation experiments with B6J mice. Open field was analyzed using two-way repeated measures ANOVA with factors of time and genotype and additional *post hoc* Bonferroni analysis. Touchscreen pairwise discrimination was analyzed using an unpaired two-tailed Student’s t-test and a χ-square test. The Grubbs test was used to identify statistical outliers using Graphpad PRISM.

### Magnetic Resonance Imaging (MRI)

A multi-channel 7.0 Tesla MRI scanner (Agilent Inc., Palo Alto, CA, USA) was used to image animals’ brains within their skulls. Sixteen custom-built solenoid coils were used to image the brains in parallel^53,54^. In order to detect volumetric changes, we used the following parameters for the MRI scan: T2- weighted, 3-D fast spin-echo sequence, with a cylindrical acquisition of k-space, a TR of 350 ms, and TEs of 12 ms per echo for 6 echoes, field-of-view equaled to 20 ×20 ×25 mm^3^ and matrix size equaled to 504 ×504 ×630. Our parameters output an image with 0.040 mm isotropic voxels. The total imaging time was 14 hours^55^.

To visualize and compare any anatomical differences in the mouse brains, the images were linearly (6 followed by 12 parameters) and non-linearly registered together. Registrations were performed with a combination of mni_autoreg tools^56^ and ANTS (advanced normalization tools^57,58^). All scans were then re-sampled with the appropriate transform and averaged to create a population atlas representing the average anatomy of the study sample. The registration results in all images was deformed into alignment with each other in an unbiased fashion. For the volume measurements, this allows for the analysis of the deformations needed to take each individual mouse’s anatomy into this final atlas space, the goal being to model how the deformation fields relate to genotype^54,59^. The Jacobian determinants of the deformation fields were then calculated as measures of volume at each voxel. Significant volume differences were calculated by warping a pre-existing classified MRI atlas onto the population atlas, which allows for the volume of 182 different segmented structures encompassing cortical lobes, large white matter structures (i.e. corpus callosum), ventricles, cerebellum, brain stem, and olfactory bulbs^60–63^ to be assessed in all brains. Further, these measurements can be examined on a voxel-wise basis in order to localize the differences found within regions or across the brain. Multiple comparisons in this study were controlled for using the false discovery rate^64^.

MWM-trained mice (WT N = 14, 7 males and 7 females; cyclin D2^−/−^ N = 13, 6 males and 7 females) and swim-stressed mice (WT N = 13, 6 males and 7 females; cyclin D2^−/−^ N = 14, 8 males and 6 females) underwent MR imaging of their brains for volumetric analyses. Hippocampal volume was defined as the sum of the volumes of CA1 oriens layer (CA1Or), CA1 pyramidal layer (CA1Py), CA1 radialis layer (CA1Rad), CA1 lacunosum moleculare layer (LMol), CA2 pyramidal layer (CA2Py), CA2 oriens layer (CA2Or), CA2 radialis layer (CA2Rad), CA3 inner pyramidal layer (CA3Py inner), CA3 outer pyramidal layer (CA3Py outer), CA oriens layer (CA3Or), CA3 radialis layer (CA3Rad), stratum lucidum (SLu), molecular layer of dentate gyrus (MoDG), granule cell layer of dentate gyrus (GrDG) and polymorph layer of dentate gyrus (PoDG). Dentate gyrus volume was defined as the sum of the volumes of molecular layer of dentate gyrus (MoDG), granule cell layer of dentate gyrus (GrDG) and polymorph layer of dentate gyrus (PoDG).

### Tissue processing and fluorescent immunohistochemistry

Animals were euthanized following the cessation of MR imaging with 4% isoflurane (whole body inhalation; to effect) and intracardially perfused with 30 mL of 0.1 M phosphate buffered saline (PBS) containing 10 U/mL heparin (Sigma) and 2 mM ProHance (a gadolinium contrast agent; Bracco Diagnostics Inc., Singen, Germany52) at 1 mL/min followed by 30 mL of 4% paraformaldehyde (PFA) in PBS containing 2 mM ProHance. Following euthanasia and fixation, brain tissue was post-fixed for 24 h in paraformaldehyde (PFA) in phosphate buffer (0.1 M Na2HPO4, 0.1 M NaH2PO4, pH 7.2) followed by equilibration in a 30% (w/v) sucrose solution in phosphate buffered saline (PBS; 3.6 mM Na2HPO4, 1.4 mM NaH2PO4, 150 mM NaCl, pH 7.2) at 4 °C. Fixed and equilibrated brain tissue was blocked in 2-mm thick coronal sections in OCT compound (Sakura Finetek, Torrance, CA) and frozen at −80 °C. Blocked brain tissue was sectioned sequentially at 10 μm in the coronal plane onto SuperFrost Plus slides (Thermo Fisher) using a cryostat (Microm HM 505E, Thermo Fisher, Waltham, MA). Slides were stored at −80 °C. Slides with sections containing the dentate gyrus (DG) and hippocampus (approximate bregma range: −1.22 mm to −2.54 mm) were thawed at room temperature, washed in PBS, and blocked in a solution containing 1% (w/v) bovine serum albumin (BSA) and 10% (v/v) normal goat serum (NGS) in 1X PBS containing 0.03% (v/v) Triton X-100.

Information regarding the antibodies and dilutions used are reported in Table 3. Sections were incubated in antibodies against Ki67 (Abcam, Cambridge, United Kingdom) and doublecortin (DCX; Millipore, Burlington, MA) overnight at 4 °C. The following day, slides were washed and incubated for 1 h at room temperature in fluorescently conjugated secondary antibodies: Alexa Fluor 488 against Ki67 primary antibody (Invitrogen, Carlsbad, CA) and Alexa Fluor 568 against DCX primary antibody (Invitrogen). Slides were mounted in ProLong Gold Antifade with 4′,6-diamidino-2-phenylindole (DAPI; Thermo Fisher) to counterstain all nuclei, coverslipped, and sealed with clear nail polish.

### Imaging and cell quantification

All imaging and cell quantification were performed by a single experimenter without knowledge of genotype or behavioral group. Immunolabeled tissue sections were visualized on a Nikon Eclipse E400 upright fluorescent microscope fitted with a SPOT RT 2.1.1 monochrome camera at 20X magnification and images of the DG were captured from both hemispheres using SPOT Advanced software (Diagnostic Instruments, Sterling Heights, MI). All cells positively labeled for Ki67 and DCX were quantified in the molecular cell layer (MCL), granule cell layer (GCL), subgranular zone (SGZ), and hilus in both hemispheres across a minimum of 3 sequential sections per brain using ImageJ (National Institutes of Health, Bethesda, MD). Double-positive labeling (Ki67^+^ DCX^+^ ) was verified by cross-referencing to the DAPI channel. Estimates of total cell populations were determined in a manner similar to what has been previously described^65^, by extrapolating raw cell counts using a sampling fraction derived from the hypothetical average regional volume divided by the total number of tissue sections sampled per animal.

### Statistical analyses for histology

Statistical analyses for histology were performed using JMP Pro (Version 14, SAS Institute, Cary, NC, USA). Histological cell count estimates were normalized to DG volume and are expressed as mean ± SEM. There was no significant difference between sexes and behavioral groups when normalized cell counts were analyzed via a multivariate ANOVA with genotype, sex, and behavior as between-group factors; therefore, sexes were combined for subsequent analyses. Normalized cell counts were then analyzed via two-way ANOVA with genotype and behavior as between-group factors, or one-way ANOVA with genotype as the between-group factor when behavioral groups are combined. *Post hoc* pairwise Student’s t-tests were performed contingent upon significant main effect (ME) from ANOVA. P values ≤ 0.05 were considered statistically significant.

Correlation analyses were performed by SAS Studio (Version 3.8, SAS Institute). Eight cyclin D2^−/−^ mice (3 males and 5 females) and 12 WT animals (6 males and 6 females) qualified for the correlation analysis as they were able to register for MR imaging and had no tissue damage after histological processing. Total estimated DCX^+^ cell counts for each animal were divided by either total hippocampal volume or total brain volume and correlated with PROC CORR to target quadrant times at 3 hours, 24 hours, 1 week, 2 weeks and 4 weeks. We built a model with PROC GLM between target quadrant times and DCX^+^ cell counts divided by total hippocampal volume or total brain volume for each time point tested and all time points averaged. The significance level for a statistically significant correlation was set at p = 0.05, determined using the F statistic.

## Supplementary information

Supplementary Information.
