# Supplementary material for: Cyclin D2-knock-out mice with attenuated dentate gyrus neurogenesis have robust deficits in long-term memory formation
Source: Sci Rep. 2020 May 18;10:8204. doi: 10.1038/s41598-020-65090-1 (PMC7235216; doi:10.1038/s41598-020-65090-1)
Supplement: Supplementary file 1 — Supplementary Information. [file 41598_2020_65090_MOESM1_ESM.pdf]

# Cyclin D2-knock-out mice with attenuated dentate gyrus neurogenesis have robust deficits in long-term memory formation

Stela P. Petkova[1&], Michael Pride[1&], Carolyn Klocke[2], Timothy A. Fenton[1], Jeannine White[3], Pamela J. Lein[2,4], Jacob Ellegood[5], Jason P. Lerch[5,6], Jill L. Silverman[1, 4], Ben Waldau[7]*

[1]Department of Psychiatry and Behavioral Sciences, University of California Davis School of Medicine, Sacramento, CA 95817
[2]Department of Molecular Biosciences, UC Davis School of Veterinary Medicine, Davis, CA 95616
[3]Institute for Regenerative Cures, Sacramento, CA 95817
[4]MIND Institute, UC Davis, Sacramento, CA 95817
[5]Mouse Imaging Centre, Hospital for Sick Children, Toronto, Ontario M5T 3H7
[6]Wellcome Centre for Integrative Neuroimaging, The University of Oxford, Oxford, OX3 9DU
[7]Department of Neurological Surgery, UC Davis Medical Center, Sacramento, CA 95817

[&] Contributed equally

**Corresponding author email address:** bwaldau@ucdavis.edu

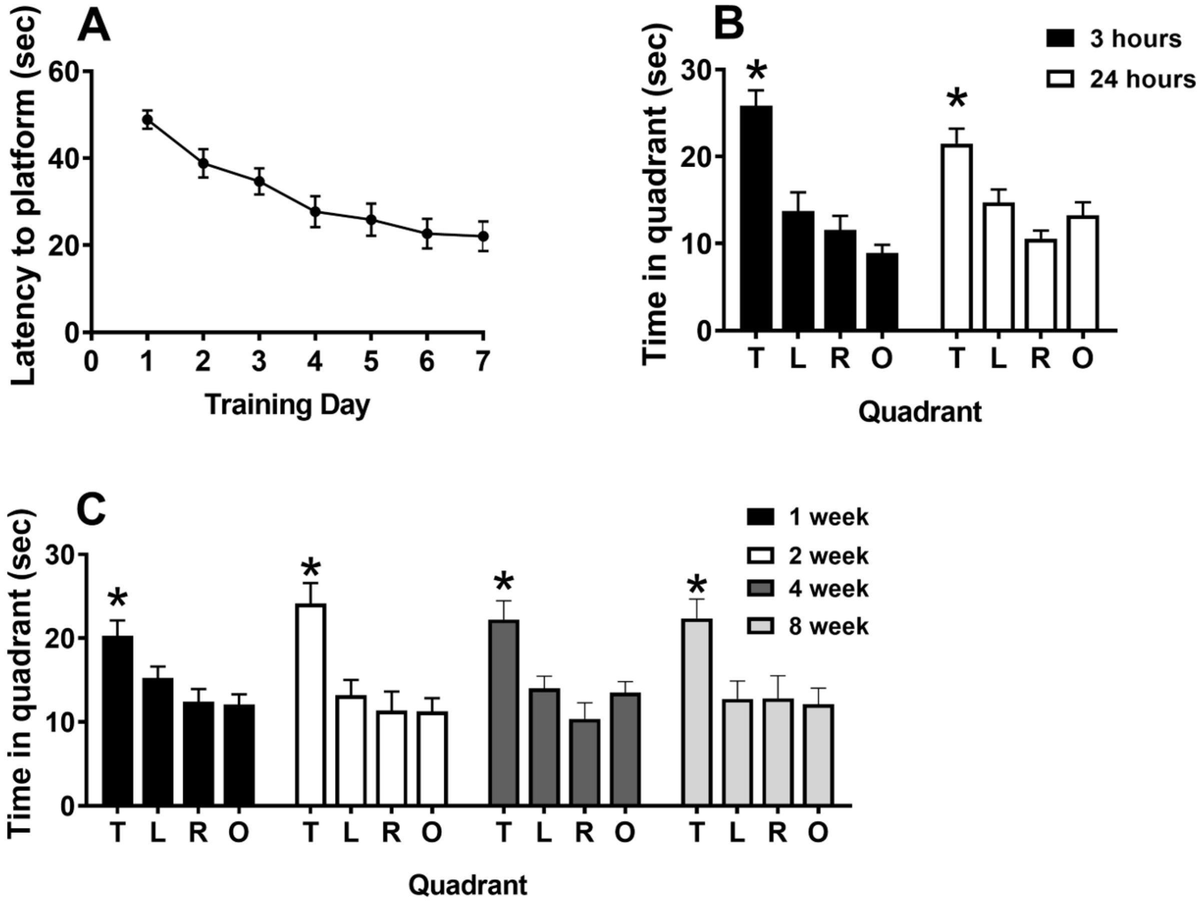


**Supplemental Figure 1**

**C57BL/6J mice demonstrate ability to retain long-term spatial memory over 8 weeks.** C57BL/6J mice (N = 16) were used to validate the modification of Morris water maze to test long-term memory over the course of several weeks. **A**, C57BL/6J mice learned where the platform was over the course of 7 training days, exhibited by a decreasing latency to find the platform. **B**, Mice show intact memory when probed at 3 hours and 24 hours after the final training session, spending significantly more time in the target quadrant compared to all others. **C**, When tested over longer intervals of 1, 2, 4 and 8 weeks, C57BL/6J mice demonstrated long-term spatial memory of the platform location. Data shown as mean ± SEM. * indicates $p < .05$ target quadrant versus other quadrants.

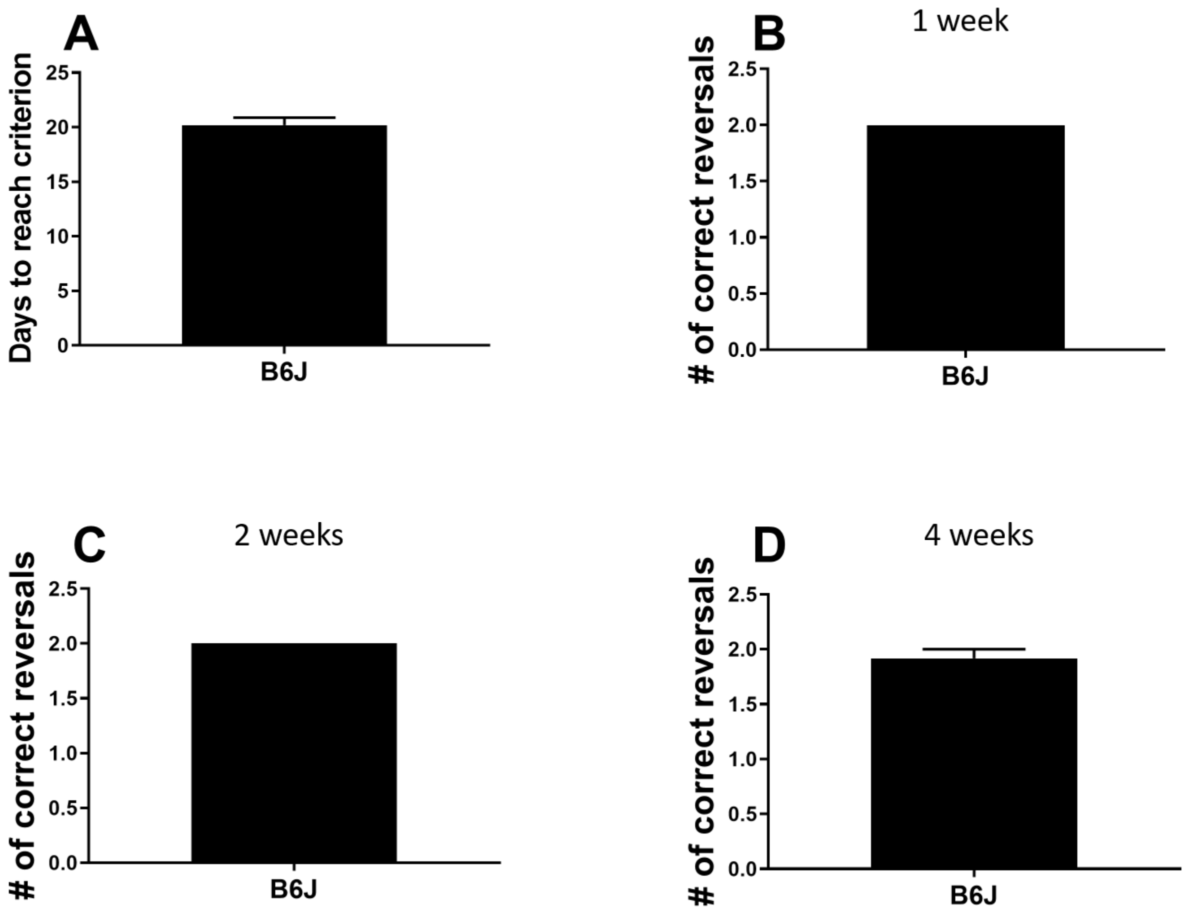


**Supplemental Figure 2**

**B6J mice demonstrate long-term memory in pattern separation task.** C57BL/6J (B6J) mice (N = 12) underwent training in an operant touchscreen assay and long-term memory of pattern separation paradigm was probed. **A,** B6J took an average of 20 daily sessions to acquire task, evaluated by the criterion of two reversals in a single session, across 3 sessions in a 4-session block. **B-D**, After the task was successfully acquired, long-term spatial memory was assessed by conducting probe trials at 1-week, 2-week and 4-week time points. Mice were tested in a single 60-minute session and the number of reversals performed was recorded. Across all time points, B6J mice are able to perform ~2 reversals during each probe session. Data shown as mean ± SEM.

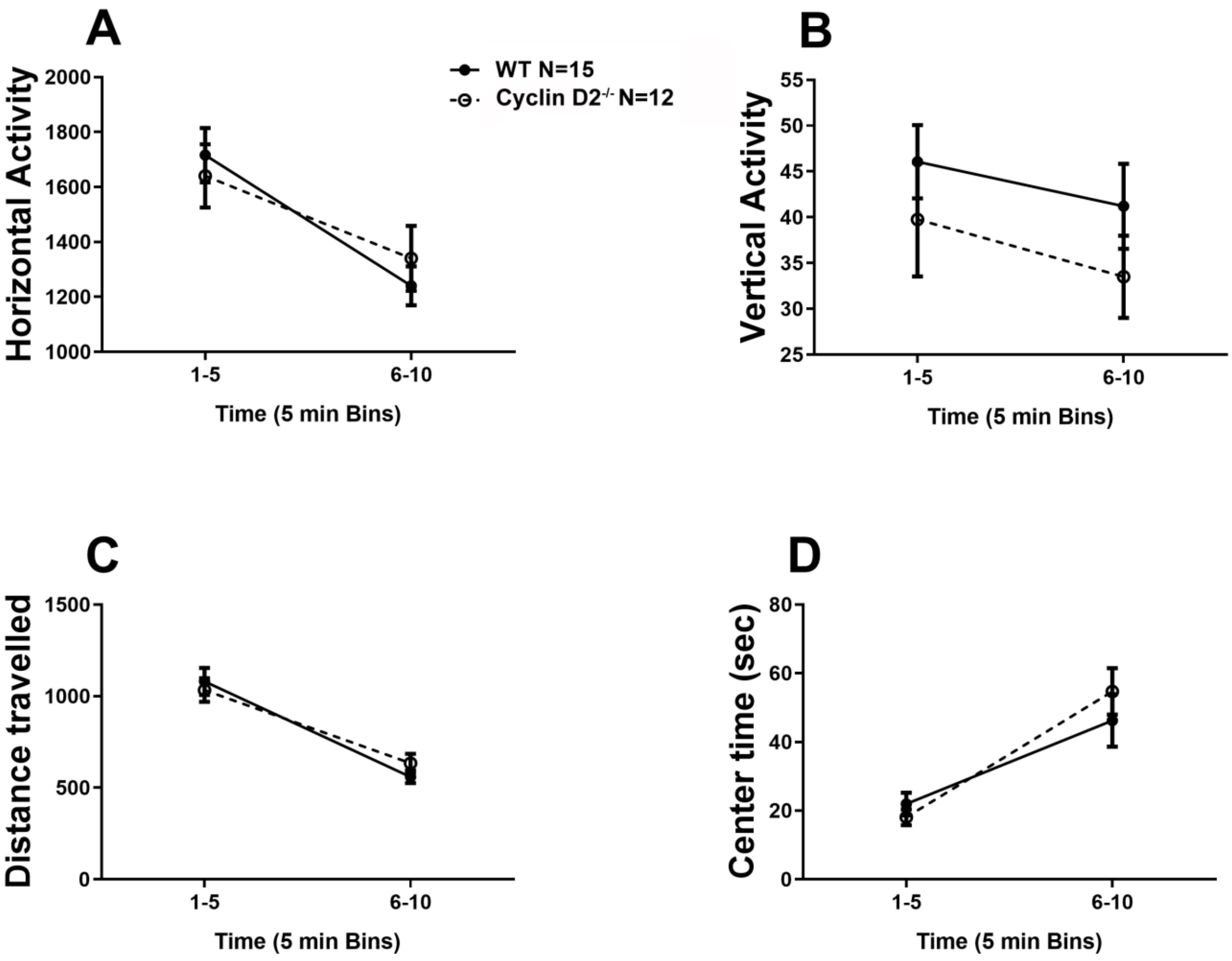


**Supplemental Figure 3**

**Cyclin D2$^{-/-}$ mice do not have altered locomotor activity. A-D**, Locomotive activity was assessed using the open field assay; mice were placed in a novel arena for 10 minutes and horizontal, vertical, total activity as well as center time was recorded. No genotype differences were found between WT (N = 15) and cyclin D2$^{-/-}$ mice (N = 12). Data shown as mean ± SEM.

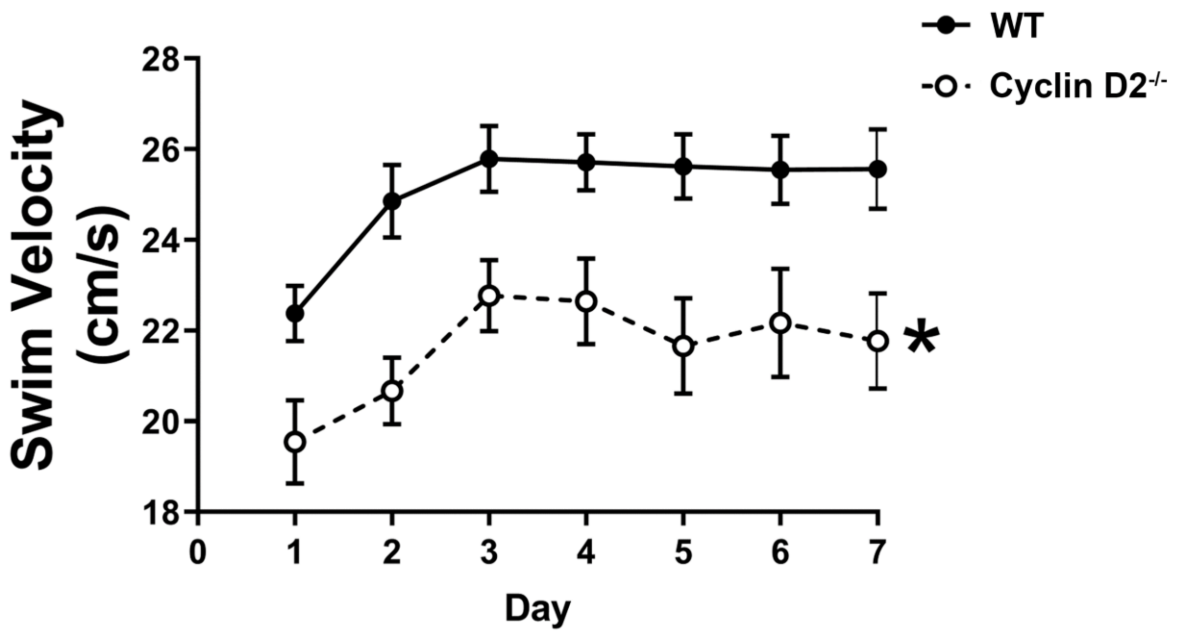


**Supplemental Figure 4**

**Cyclin D2$^{-/-}$ mice showed decreased swim velocity in acquisition of Morris water maze task.** Swim velocity was recorded during water maze training sessions, and cyclin D2$^{-/-}$ mice (N = 27) demonstrated slower swim speeds compared to WT (N = 20). Data shown as mean ± SEM. * indicates $p < .05$, main effect of genotype in repeated measures two-way ANOVA.

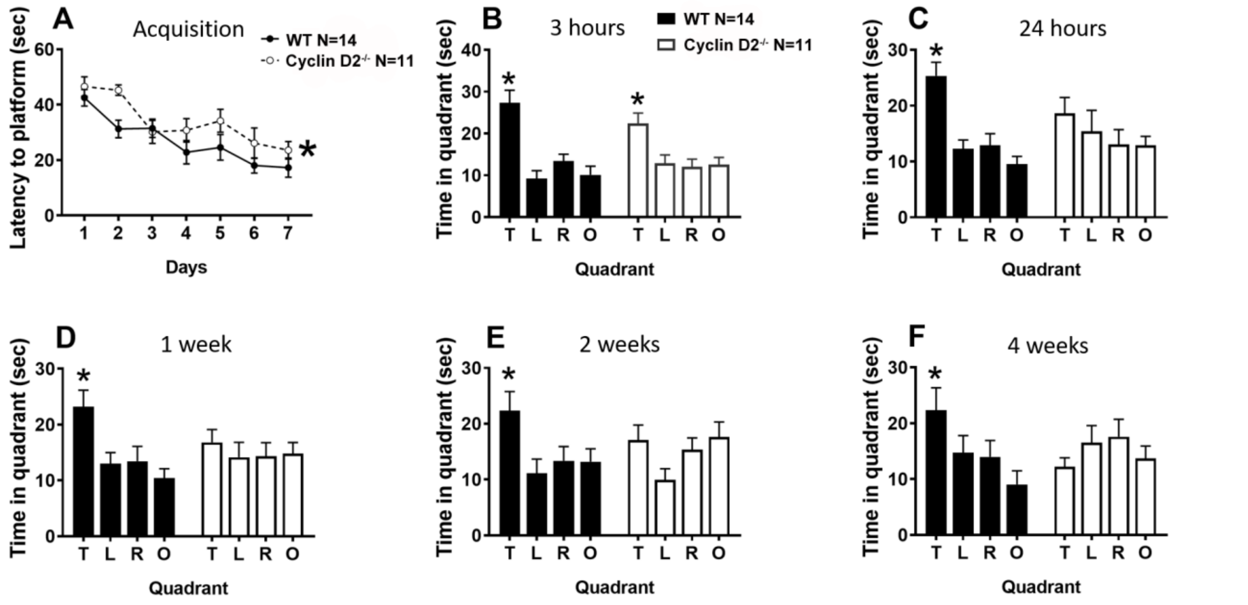


**Supplemental Figure 5**

**Independent cohort replicates long-term spatial memory deficit in cyclin D2$^{-/-}$ mice. A**, Cyclin D2$^{-/-}$ mice (N = 11) were able to acquire the location of the platform over the course of 7 training days. WT mice (N = 14), again, performed significantly better in latency to platform. **B**, Both WT and cyclin D2$^{-/-}$ had intact short-term memory during a probe trial 3 hours after the last training session. **C**, At 24 hours, cyclin D2$^{-/-}$ showed a deficit in spatial memory of the platform location. WT demonstrated intact memory by spending significantly more time in the target quadrant compared to the other quadrants. **D-F**, Cyclin D2$^{-/-}$ mice did not show intact long-term memory of the platform location when probed at 1-week, 2-weeks, and 4-weeks, whereas WT do show intact long-term memory of the platform location. Data shown as mean ± SEM. * indicates $p < .05$ target quadrant versus other quadrants.

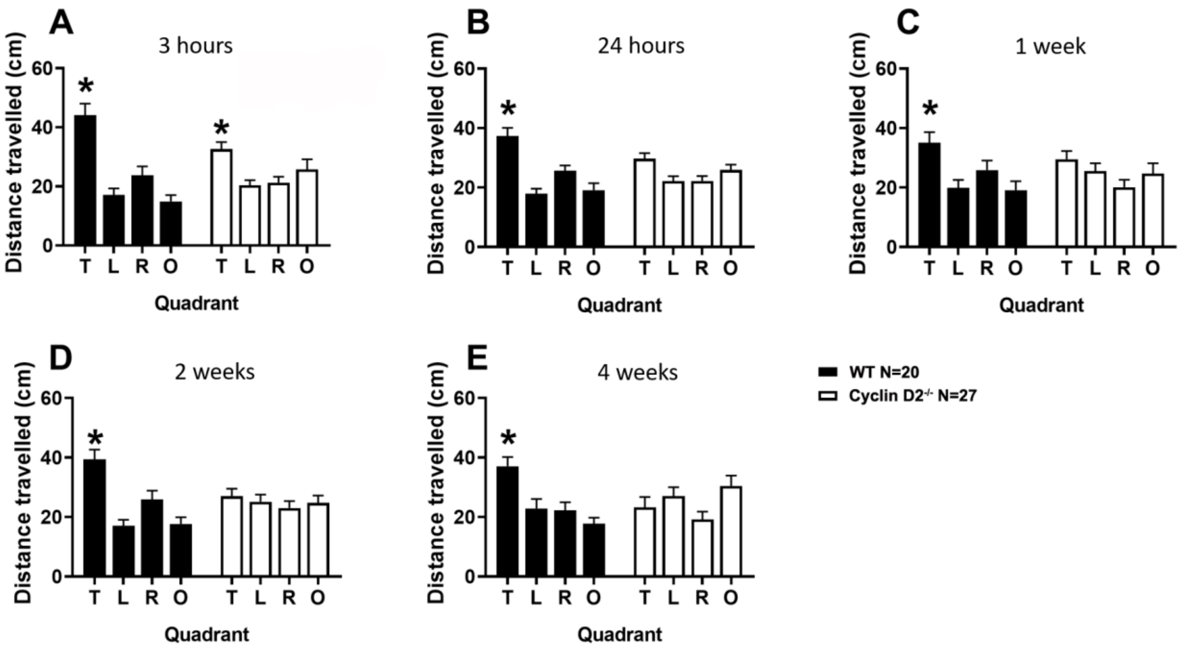


**Supplemental Figure 6**

**Cyclin D2$^{-/-}$ mice demonstrate long-term memory deficits in the Morris water maze when slower swim speed is accounted for.** To ensure that phenotype observed was not due to reduced swim speed, Morris water maze data was analyzed by looking at the distance travelled within each quadrant. **A,** Three hours after the last training session, WT and cyclin D2$^{-/-}$ mice both swam around more in the target quadrant compared to the other quadrants, suggesting both groups had intact short-term memory. **B-E**, The long-term spatial memory deficits cyclin D2$^{-/-}$ mice displayed were reinforced in this analysis; WT travelled more in the target quadrant at the 24-hours, 1-week, 2-week and 4-week probes compared to the cyclin D2$^{-/-}$ mice. Data shown as mean ± SEM. * indicates $p < .05$ target quadrant versus other quadrants.

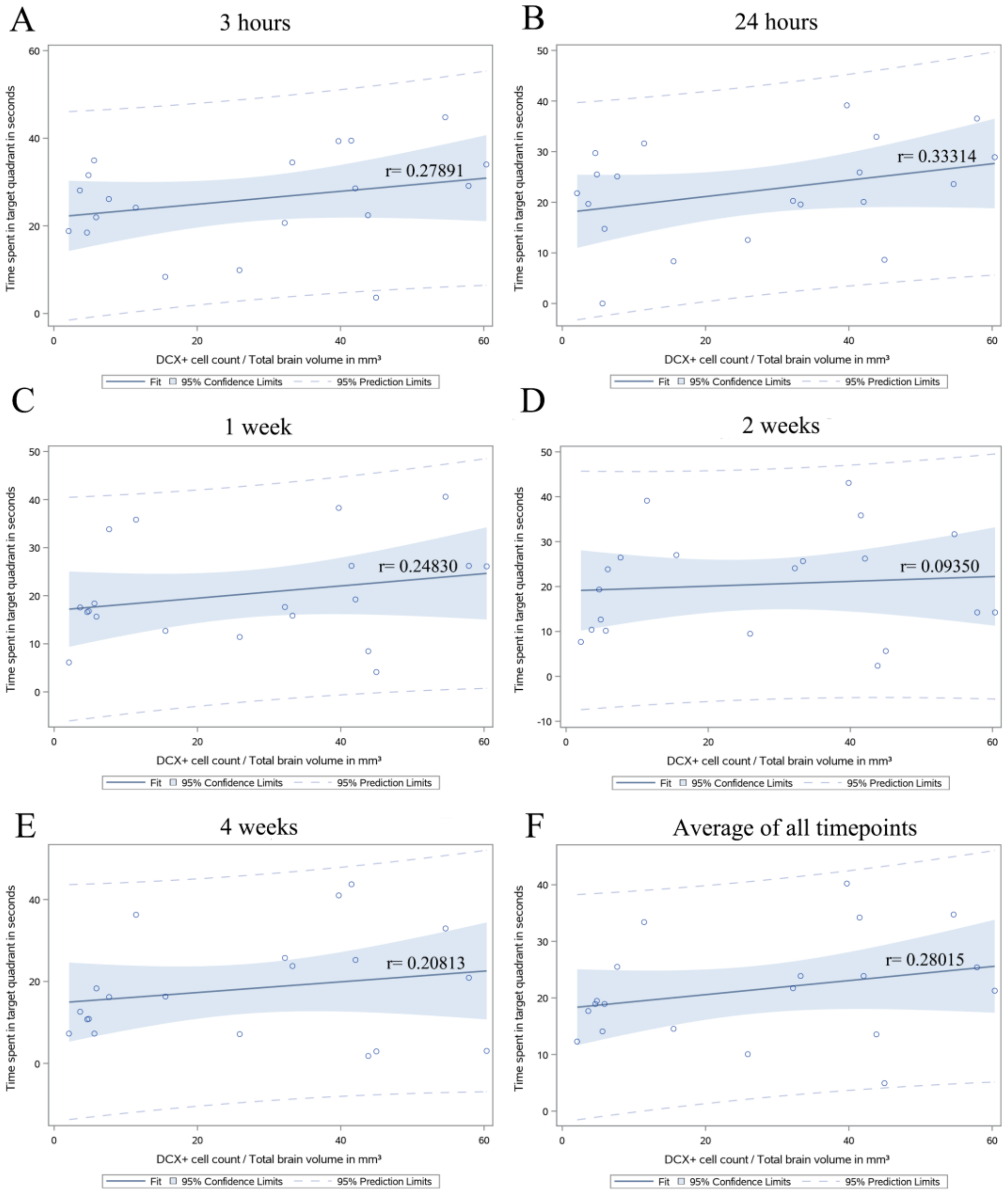


**Supplemental Figure 7**

**Dentate neurogenesis adjusted for differences in total brain volume moderately correlates with time spent in the target quadrant 24 hours after Morris water maze acquisition learning. A**, A weak correlation was found between the number of DCX+ cells normalized to total brain volume and time spent in target quadrant 3 hours after acquisition. **B,** A moderate correlation (r= 0.33314, $F_{(1,18)}$ = 2.25, p= 0.1512) between DCX+ cell count adjusted for total brain volume and time spent in target quadrant was seen 24 hours after acquisition. **C-F**, The correlation between DCX+ cell count adjusted for total brain volume was weaker at later time points and across all tested time points.

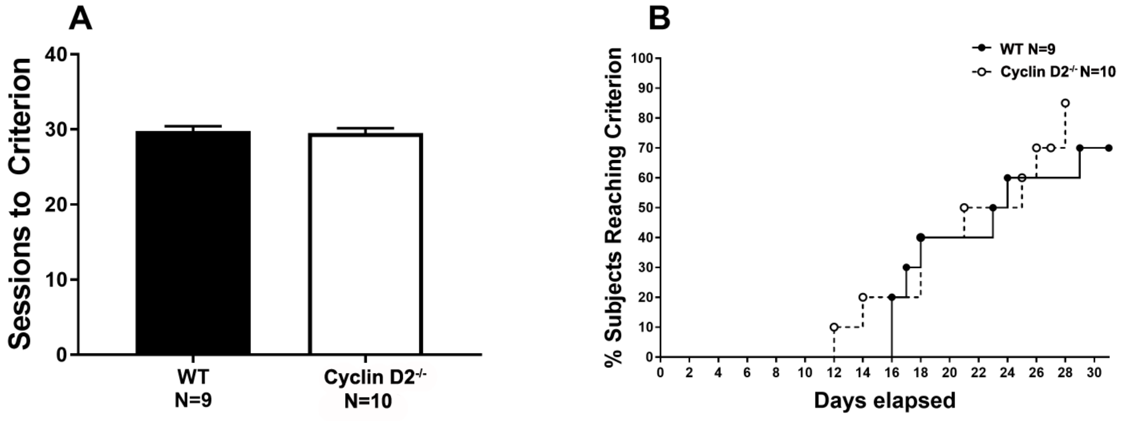


**Supplemental Figure 8**

**Cyclin D2$^{-/-}$ mice have intact visual discrimination in a non-hippocampal pairwise touchscreen assay. A**, Subjects were trained in operant touchscreen chambers and then a non-hippocampal, non-spatial task was employed. WT (N = 9) and cyclin D2$^{-/-}$ mice (N = 10) acquired the task in a similar number of sessions, reaching the criterion of 80% accuracy in two consecutive days in an average of 29 sessions. **B**, The proportion of individual subjects that completed acquisition at each training day (survival curve) was not significantly different between genotype. Data shown as mean ± SEM.

**Supplemental Table 1**

Statistical data of Supplemental Figures

| Figure | Group | Metric | One Way ANOVA | | Bonferroni Post Hoc | | |
|---|---|---|---|---|---|---|---|
| | | | F Statistic | p value | T vs. L | T vs. R | T vs. O |
| S1, B | C57BL/6J N=16 | 3-hr Probe, Time in Quadrant | F (3,60) = 19.98 | **p < .0001*** | **p < .0001*** | **p < .0001*** | **p < .0001*** |
| S1, B | | 24-hr Probe, Time in Quadrant | F (3,60) = 10.58 | **p < .0001*** | **p = .0043*** | **p < .0001*** | **p = 0004*** |
| S1, C | | 1-week Probe, Time in Quadrant | F (3,60) = 6.504 | **p = .0007*** | **p = .0568†** | **p = .0013*** | **p = .0007*** |
| S1, C | | 2-week Probe, Time in Quadrant | F (3,60) = 9.003 | **p < .0001*** | **p = .0012*** | **p = .0001*** | **p = .0001*** |
| S1, C | | 4-week Probe, Time in Quadrant | F (3,60) = 8.133 | **p = .0001*** | **p = .0054*** | **p < .0001*** | **p = .0028*** |
| S1, C | | 8-week Probe, Time in Quadrant | F (3,60) = 4.623 | **p = .0056*** | **p = .0126*** | **p = .0132*** | **p = .0072*** |

| Figure Panel | Group | Metric | Mean | SEM | Range |
|---|---|---|---|---|---|
| S2, A | C57BL/6J N=12 | Days to Criterion | 20.20 | 0.6799 | 18-24 |
| S2, B | | # of correct reversals, 1-week probe | 2 | 0 | |
| S2, C | | # of correct reversals 2-week probe | 2 | 0 | |
| S2, D | | # of correct reversals 4-week probe | 1.917 | 0.08333 | 1-2 |

| Figure Panel | Test | Sample Size | Metric | Time Point | Statistical Test | Statistic | p value | *posthoc test* | p value |
|---|---|---|---|---|---|---|---|---|---|
| S3, A | Open Field | WT N=15, Cyclin D2$^{-/-}$ N=12 | Horizontal Activity Over Time | 1-5 | Two Way Repeated Measures ANOVA | Genotype F (1, 25) = 0.0099<br>Time F (1, 25) = 30.71<br>Interaction F (1, 25) = 1.578 | p =.9215<br>**p < .0001***<br>p =.2207 | WT vs. Cyclin D2$^{-/-}$ | p > .9999 |
| | | | | 6-10 | | | | | p = .9642 |
| S3, B | | | Vertical Activity Over Time | 1-5 | Two Way Repeated Measures ANOVA | Genotype F (1, 25) = 1.488<br>Time F (1, 25) = 2.226<br>Interaction F (1, 25) = 03446 | p =.2339<br>p =.1482<br>p =.8542 | WT vs. Cyclin D2$^{-/-}$ | p = .7214 |
| | | | | 6-10 | | | | | p = .5323 |

| | | | | | | | | | |
|---|---|---|---|---|---|---|---|---|---|
| S3, C | | | Total Activity Over Time | 1-5 | Two Way Repeated Measures ANOVA | Genotype F (1, 25) = .0401<br>Time F (1, 25) = 94.72<br>Interaction F (1, 25) = 1.652 | p =.8429<br>**p <.0001***<br>p =.2105 | WT vs. Cyclin D2$^{-/-}$ | p > .9999 |
| | | | | 6-10 | | | | | p = .7478 |
| S3, D | | | Center Time over Time | 1-5 | Two Way Repeated Measures ANOVA | Genotype F (1, 25) = 0.1327<br>Time F (1, 25) = 39.51<br>Interaction F (1, 25) = 1.620 | p = .7188<br>**p <.0001***<br>p =.2148 | WT vs. Cyclin D2$^{-/-}$ | p > .9999 |
| | | | | 6-10 | | | | | p = .5851 |

| **Figure** | **Test** | **Sample Size** | **Metric** | **Time Point** | **Statistical Test** | **Statistic** | **p value** | **posthoc test** | **p value** |
|---|---|---|---|---|---|---|---|---|---|
| S4 | Acquisition of Morris Water Maze | WT N=20, Cyclin D2$^{-/-}$ N=27 | Swim Velocity | Day 1 | Two Way Repeated Measures ANOVA | Day F (6,270) = 6.318<br>Genotype F (1,45) = 12.8<br>Interaction F (6,270) = 0.3247 | **p<.0001***<br>**p =.0008***<br>p =.9237 | Bonferroni WT vs. Cyclin D2$^{-/-}$ | p = .2010 |
| | | | | Day 2 | | | | | **p = .0090*** |
| | | | | Day 3 | | | | | p = .1407 |
| | | | | Day 4 | | | | | p = .1268 |
| | | | | Day 5 | | | | | **p = .0160*** |
| | | | | Day 6 | | | | | p = .0642† |
| | | | | Day 7 | | | | | **p = .0248*** |

| **Figure** | **Test** | **Sample Size** | **Metric** | **Time Point** | **Statistical Test** | **Statistic** | **p value** | **posthoc test** | **p value** |
|---|---|---|---|---|---|---|---|---|---|
| S5, A | Acquisition of Morris Water Maze | WT N=14, Cyclin D2$^{-/-}$ N=11 | Latency to Platform | Day 1 | Two Way Repeated Measures ANOVA | Day F (6,138) = 11.91<br>Genotype F (1,23) = 6.941<br>Interaction F (6,138) = 0.9106 | **p<.0001***<br>**p =.0148***<br>p =.4893 | Bonferroni WT vs. Cyclin D2$^{-/-}$ | p > .9999 |
| | | | | Day 2 | | | | | p=.0656† |
| | | | | Day 3 | | | | | p > .9999 |
| | | | | Day 4 | | | | | p=.9636 |
| | | | | Day 5 | | | | | p=.5086 |
| | | | | Day 6 | | | | | p = .9255 |
| | | | | Day 7 | | | | | p > .9999 |

| **Figure** | **Metric** | **Group** | **One Way ANOVA** | | **Bonferroni Post Hoc** | | |
|---|---|---|---|---|---|---|---|
| | | | **F Statistic** | **p value** | **T vs. L** | **T Vs. R** | **T vs. O** |
| S5, B | 3-hr Probe, Time in Quadrant | WT | F (3, 52) = 14.25 | **p < .0001*** | **p < .0001*** | **p = .0001*** | **p < .0001*** |
| | | Cyclin D2$^{-/-}$ | F (3, 40) = 6.281 | **p = .0014*** | **p = .0047*** | **p = .0020*** | **p = .0032*** |
| S5, C | 24-hr Probe, | WT | F (3, 52) = 13.36 | **p < .0001*** | **p < .0001*** | **p < .0001*** | **p < .0001*** |

| | | | | | | | |
|---|---|---|---|---|---|---|---|
| | Time in Quadrant | Cyclin D2$^{-/-}$ | F (3, 40) = 0.9229 | p = .4385 | p > .9999 | p = .5001 | p = .4596 |
| S5, D | 1-week Probe, Time in Quadrant | WT | F (3, 52) = 5.632 | **p = .0020*** | **p = .0111*** | **p = .0156*** | **p = .0011*** |
| | | Cyclin D2$^{-/-}$ | F (3, 40) = 0.2668 | p = .8490 | p > .9999 | p > .9999 | p > .9999 |
| S5, E | 2-week Probe, Time in Quadrant | WT | F (3, 52) = 3.345 | **p = .0260*** | **p = .0161** | p = .0721† | p = .0654† |
| | | Cyclin D2$^{-/-}$ | F (3, 40) = 2.198 | p = .1032 | p = .1153 | p > .9999 | p > .9999 |
| S5, F | 4-week Probe, Time in Quadrant | WT | F (3, 52) = 2.988 | **p = .0393*** | p = .2907 | p = .2070 | **p = .0139*** |
| | | Cyclin D2$^{-/-}$ | F (3, 40) = 0.9332 | p = .4336 | p = .7252 | p = .4433 | p > .9999 |

| **Figure** | **Metric** | **Group** | **One Way ANOVA** | | **Bonferroni Post Hoc** | | |
|---|---|---|---|---|---|---|---|
| | | | **F Statistic** | **p value** | **T vs. L** | **T Vs. R** | **T vs. O** |
| S6, A | 3-hr Probe, Distance travelled | WT | F (3, 76) = 21.38 | **p < .0001*** | **p < .0001*** | **p < .0001*** | **p < .0001*** |
| | | Cyclin D2$^{-/-}$ | F (3, 104) = 5.060 | **p = .0026*** | **p = .0022*** | **p = .005*** | p = .1617 |
| S6, B | 24-hr Probe, Distance travelled | WT | F (3, 76) = 16.55 | **p < .0001*** | **p < .0001*** | **p = .0009*** | **p < .0001*** |
| | | Cyclin D2$^{-/-}$ | F (3, 104) = 4.179 | **p = .0078*** | **p = .0093*** | **p = .0097*** | p = .3933 |
| S6, C | 1-week Probe, Distance travelled | WT | F (3, 76) = 5.634 | **p = .0015*** | **p = .0026*** | p = .1168 | **p = .0015*** |
| | | Cyclin D2$^{-/-}$ | F (3, 104) = 1.857 | p = .1415 | p = .9784 | p = .0623 | p = .7020 |
| S6, D | 2-week Probe, Distance travelled | WT | F (3, 76) = 15.04 | **p < .0001*** | **p < .0001*** | **p = .0019*** | **p < .0001*** |
| | | Cyclin D2$^{-/-}$ | F (3, 104) = 0.4573 | p = .7127 | p > .9999 | p = .7383 | p > .9999 |
| S6, E | 4-week Probe, Distance travelled | WT | F (3, 76) = 9.032 | **p < .0001*** | **p = .0016*** | **p = .0010*** | **p < .0001*** |
| | | Cyclin D2$^{-/-}$ | F (3, 104) = 2.421 | p = .0702† | p > .9999 | p > .9999 | p = .3215 |

| **Figure** | **Test** | **Sample Size** | **Metric** | **Statistical Test** | **Statistic** | **P value** |
|---|---|---|---|---|---|---|
| S8, A | Touchscreen-Pairwise Discrimination | WT N=9, Cyclin D2$^{-/-}$ N=10 | Sessions to Criteria | Unpaired Two-Tailed T-Test | T (17) = 0.2937 | p = .7726 |
| S8, B | | | % Animals Completed | Gehan Breslow Wilcoxon Chi Square Test | X = 0.1463 | p = .7021 |
